# Supplementary material for: Modeling human telencephalic development and autism-associated SHANK3 deficiency using organoids generated from single neural rosettes
Source: Nat Commun. 2022 Oct 6;13:5688. doi: 10.1038/s41467-022-33364-z (PMC9537523; doi:10.1038/s41467-022-33364-z)
Supplement: Supplementary file 1 — Supplementary Information [file 41467_2022_33364_MOESM1_ESM.pdf]

Supplementary Figures and Tables

Modeling human telencephalic development and autism-associated SHANK3 deficiency using organoids generated from single neural rosettes

Yueqi Wang[1,2], Simone Chiola[1], Guang Yang[1,2], Chad Russell[3], Celeste J. Armstrong[1], Yuanyuan Wu[1], Jay Spampanato[4], Paisley Tarboton[3], H. M. Arif Ullah[1], Nicolas U. Edgar[1], Amelia N. Chang[5], David A. Harmin[5], Vittoria Dickinson Bocchi[6,7], Elena Vezzoli[6,7], Dario Besusso[6,7], Jun Cui[8], Elena Cattaneo[6,7], Jan Kubanek[3], Aleksandr Shcheglovitov[1,2,3]

[1]Department of Neurobiology, University of Utah, Salt Lake City, UT, USA

[2]Neuroscience Graduate Program, University of Utah, Salt Lake City, UT, USA

[3]Department of Biomedical Engineering, University of Utah, Salt Lake City, UT, USA

[4]Department of Neurosurgery, University of Utah, Salt Lake City, UT, USA

[5]Department of Neurobiology, Harvard Medical School, Boston, MA, USA

[6]Department of Biosciences, University of Milan, Milan, Italy

[7]Istituto Nazionale di Genetica Molecolare, Milan, Italy

[8]Department of Cell Biology and Neurosciences, Montana State University, Bozeman, MT, USA

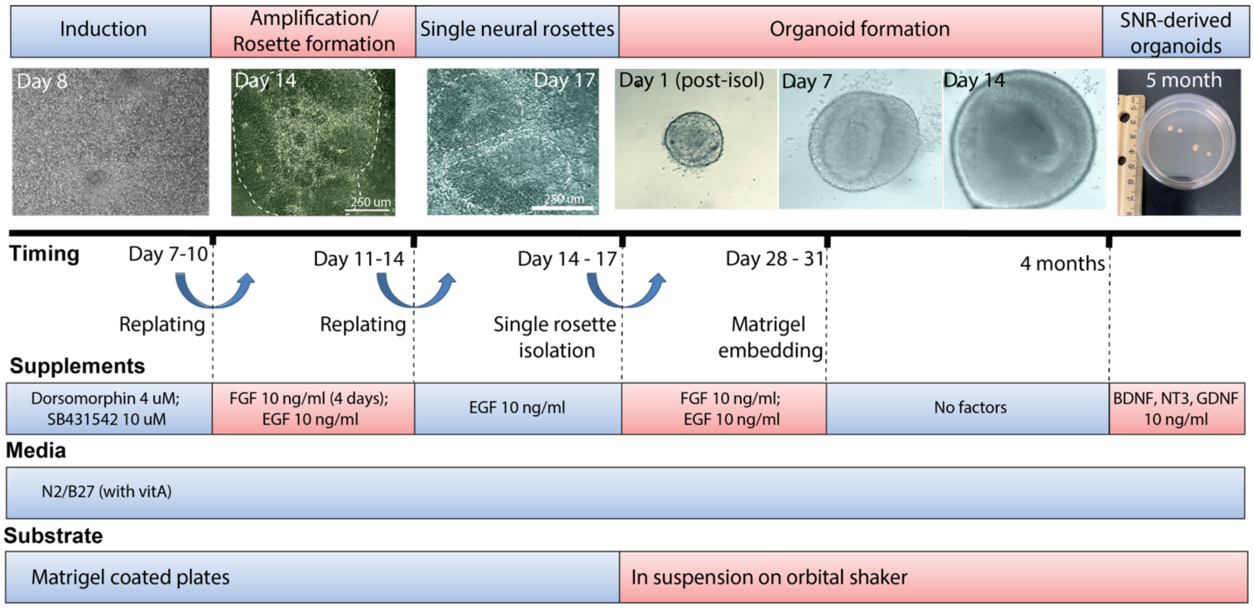

Supplementary Figure 1. Protocol to produce SNR-derived organoids

Dual SMAD inhibitors (Dorsomorphin and SB431542) are used to convert pluripotent stem cells into neuroepithelial-like cells (Induction). FGF and EGF are used to amplify the population of neural progenitors and to induce the formation of neural rosettes (Amplification/Rosette formation). Sing Neural Rosettes (SNR) (200-250 μm in diameter) are isolated 14-17 days post induction (Single neural rosettes) and cultured in 1:1 mixture of N2 and B27 (with vitamin A) media supplemented with EGF and FGF on an orbital shaker in an incubator (5% CO_2 and 37°C) (Organoid formation). 28-31 days post-induction organoids are embedded in Matrigel and cultured on orbital shaker in N2/B27 (with vitamin A) media without growth or trophic factors. 4 months post-induction, trophic factors are added to the media to promote functional and synaptic neuronal maturation (SNR-derived organoids). Additional protocol details are provided in the Methods section.

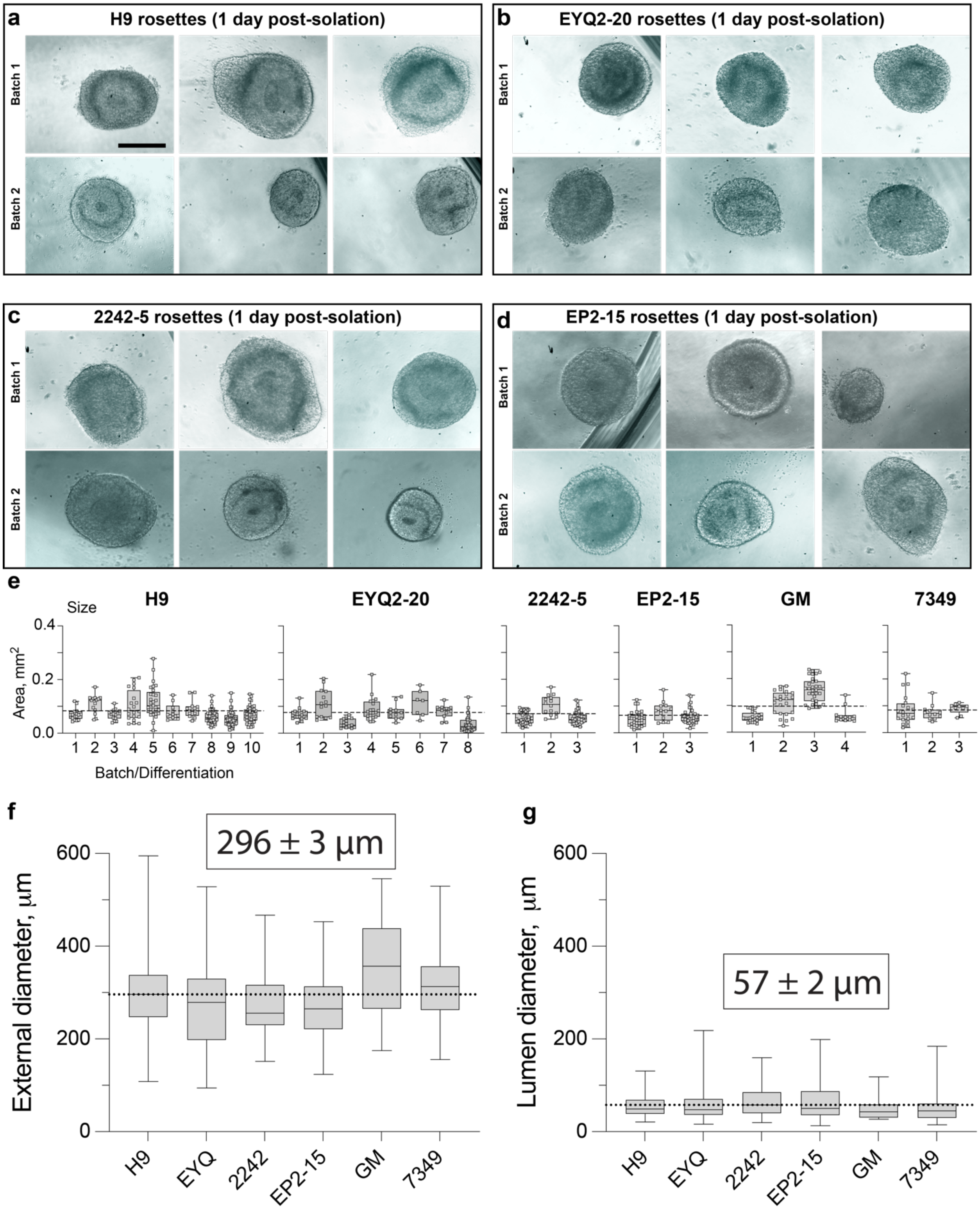
a
H9 rosettes (1 day post-solation)
Batch 1
Batch 2
b
EYQ2-20 rosettes (1 day post-solation)
Batch 1
Batch 2
c
2242-5 rosettes (1 day post-solation)
Batch 1
Batch 2
d
EP2-15 rosettes (1 day post-solation)
Batch 1
Batch 2
e
Size
H9
EYQ2-20
2242-5
EP2-15
GM
7349
Area, mm2
0.4
0.2
0.0
Batch/Differentiation
f
296 ± 3 µm
External diameter, µm
600
400
200
0
H9
EYQ
2242
EP2-15
GM
7349
g
57 ± 2 µm
Lumen diameter, µm
600
400
200
0
H9
EYQ
2242
EP2-15
GM
7349

Supplementary Figure 2. Characterization of isolated SNRs.

a-d, Representative images of SNRs produced from different pluripotent stem cell lines in two differentiation batches at day 1 post-isolation. **e,** Quantification of SNR size across multiple differentiation batches for different stem cell lines (n = 14 [batch1], 14 [batch2], 16 [batch3], 20 [batch4], 29 [batch5], 10 [batch6], 12 [batch7], 47 [batch8], 58 [batch9], and 40 [batch10]; 14 [batch1], 14 [batch2], 15 [batch3], 20 [batch4], 11 [batch5], 7 [batch6], 14 [batch7], and 40 [batch8]; 39 [batch1], 14 [batch2], and 42 [batch3]; 30 [batch1], 13 [batch2], and 38 [batch3]; 24 [batch1], 23 [batch2], 32 [batch3], and 12 [batch4]; and 20 [batch1], 12 [batch2], and 9 [batch3] organoids produced from H9, EYQ2-20, 2242-5, EP2-15, GM, and 7349-5 lines, respectively). **f-g,** Quantification of external (n = 260, 132, 95, 81, 91, and 41 organoids produced in 3-10 differentiation batches from H9, EYQ2-20, 2242-5, EP2-15, GM, and 7349-5 lines, respectively) (**e**) and lumen diameter (n = 53 [H9], 56 [EYQ2-20], 16 [2242-5], 27 [EP2-15], 20 [GM], and 21 [7349-5] organoids produced in 3-10 differentiation batches from H9, EYQ2-20, 2242-5, EP2-15, GM, and 7349-5 lines, respectively) (**e**) of SNRs produced from different stem cell lines. Data presented as individual data point and box plots (median ± $25^{th}/75^{th}$ [box] and min and max values [whiskers]) (**e**) and box plots (median ± $25^{th}/75^{th}$ [box] and min and max values [whiskers]). Scale bars = 250 µm. Source data are provided as a Source Data file.

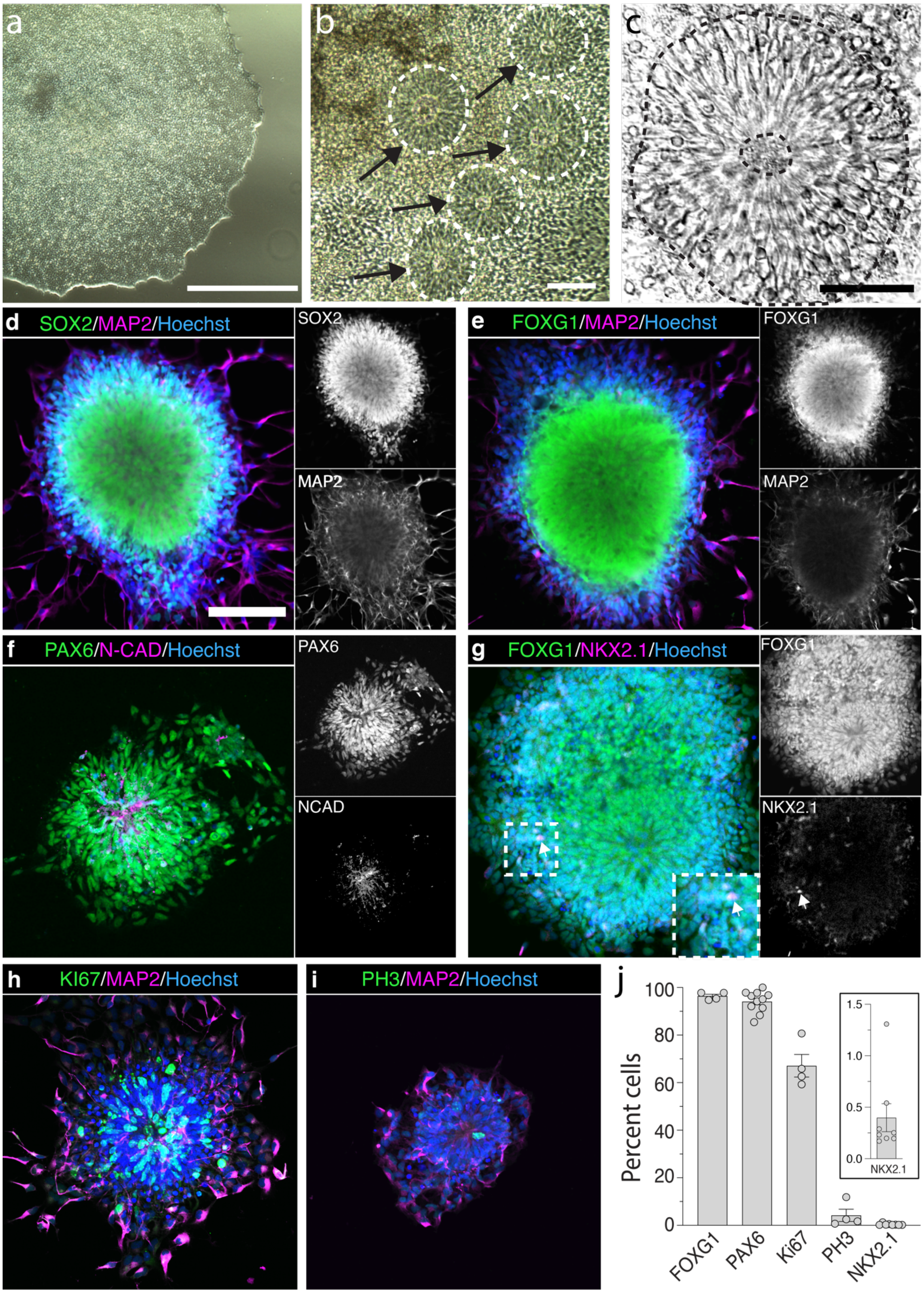
a
b
c
d
SOX2/MAP2/Hoechst
SOX2
MAP2
e
FOXG1/MAP2/Hoechst
FOXG1
MAP2
f
PAX6/N-CAD/Hoechst
PAX6
NCAD
g
FOXG1/NKX2.1/Hoechst
FOXG1
NKX2.1
h
KI67/MAP2/Hoechst
i
PH3/MAP2/Hoechst
j
Percent cells
100
80
60
40
20
0
FOXG1
PAX6
Ki67
PH3
NKX2.1
1.5
1.0
0.5
0.0
NKX2.1

Supplementary Figure 3. Isolation and characterization of human stem cell-derived SNRs

a-b, Low magnification images of an iPSC colony (**a**) and cluster of iPSC-derived SNRs (**b**). **c**, High magnification image of an isolated SNR. **d-h**, Images of isolated SNRs immunostained with antibodies against SOX2 and MAP2 (**d**), FOXG1 and MAP2 (**e**), PAX6 and N-Cad (**f**), PAX6 and N-Cad (**g**), Ki67 and MAP2 (**h**), and PH3 and MAP2 (**i**). **j**, Percentages of cells expressing different markers in SNRs (n = 4 [FOXG1], 11 [PAX6], 4 [Ki67], 4 [PH3] and 8 [NKX2-1] organoids produced in 2–4 batches from 2242-5, EP2-15, H9, and EYQ2-20 lines). Data presented as means ± s.e.m. Scale bars = 1000 (**a**), 200 (**b**), 50 (**c**), and 100 μm (**d**). Source data are provided as a Source Data file.

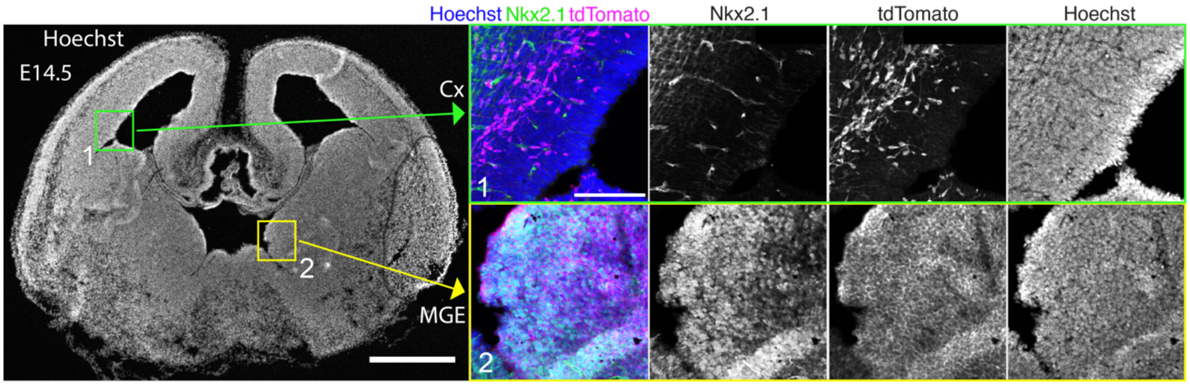

Supplementary Figure 4. NKX2.1 expression in developing mouse brain.

Image of a coronal brain section from a conditional $Rosa^{tdTomato}/Nkx2.1^{Cre}$ mouse at E14.5 (Xie et al., Plos Biol., 2017)[1] immunostained with antibodies against Nkx2.1 to validate anti-Nkx2.1 antibody specificity. Nkx2.1 expression was detected in all cells in MGE (yellow box, green nuclear signal, bottom panel 2) where it overlapped with tdTomato (red cytoplasmic signal, bottom panel 2). Nkx2.1 expression was not detected in migrating MGE-derived interneurons expressing tdTomato in the pallium (green box, top panel 1). Scale bars = 500 and 100 µm.

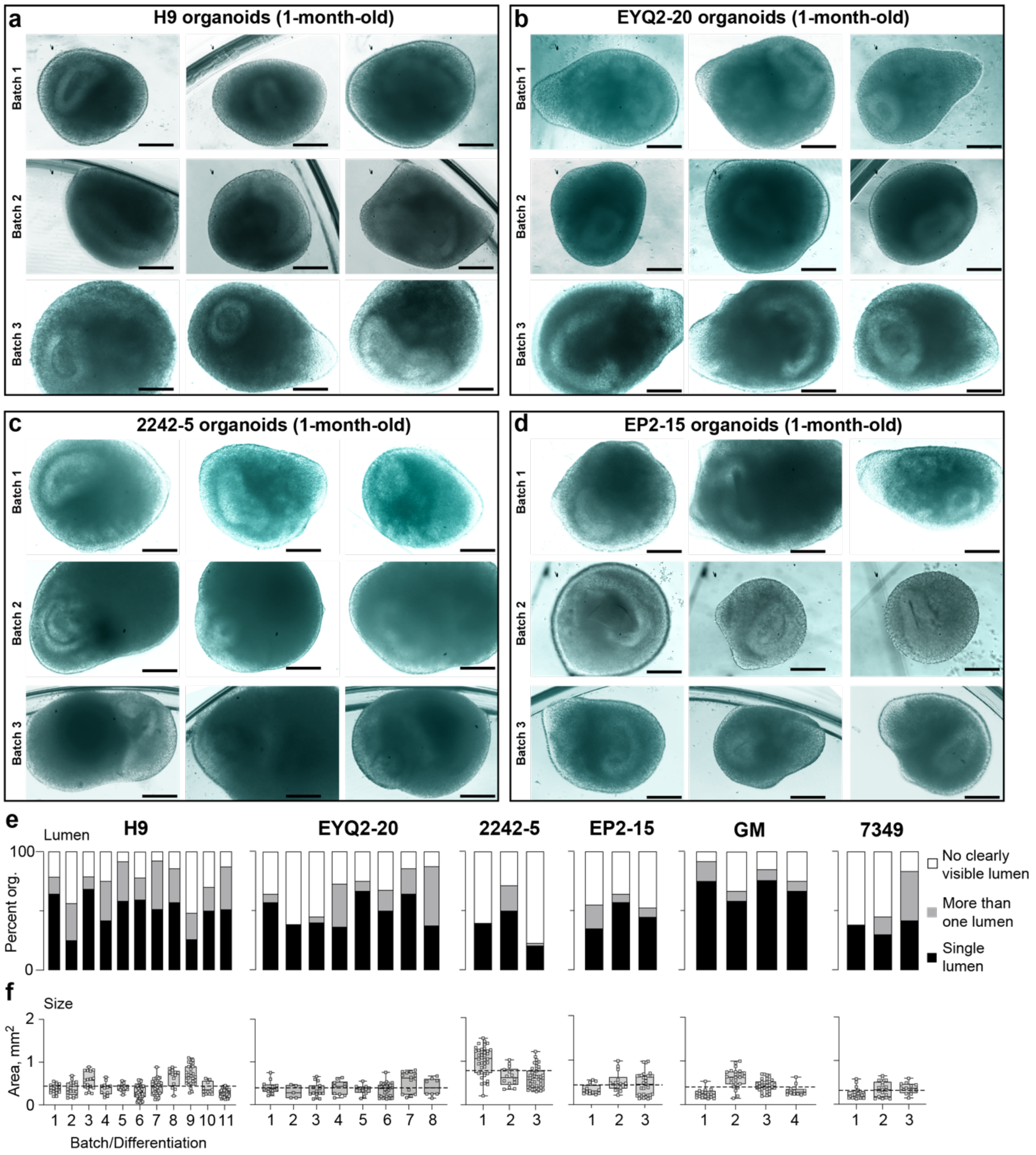

Supplementary Figure 5. Characterization of 1-month-old SNR-derived organoids.

a-d, Representative images of SNR-derived organoids produced from different pluripotent stem cell lines in three differentiation batches. **e-f,** Quantification of lumen presence (**e**) and organoid size (**f**) across multiple differentiation batches for different stem cell lines (n = 14 [batch1], 16 [batch2], 17 [batch3], 12 [batch4], 12 [batch5], 59 [batch6], 38 [batch7], 15 [batch8], 27 [batch9], 9 [batch10] and 43 [batch11]; 12 [batch1], 5 [batch2], 15 [batch3], 10 [batch4], 12

[batch5], 30 [batch6], 14 [batch7], and 8 [batch8]; 38 [batch1], 14 [batch2], and 42 [batch3]; 13 [batch1], 14 [batch2], and 29 [batch3]; 16 [batch1], 24 [batch2], 32 [batch3], and 12 [batch4]; and 21 [batch1], 18 [batch2], and 12 [batch3] organoids produced from H9, EYQ2-20, 2242-5, EP2-15, GM, and 7349-5 lines, respectively). Data presented as individual data points and box plots (median ± $25^{th}/75^{th}$ [box] and min and max values [whiskers]). Scale bars = 250 µm. Source data are provided as a Source Data file.

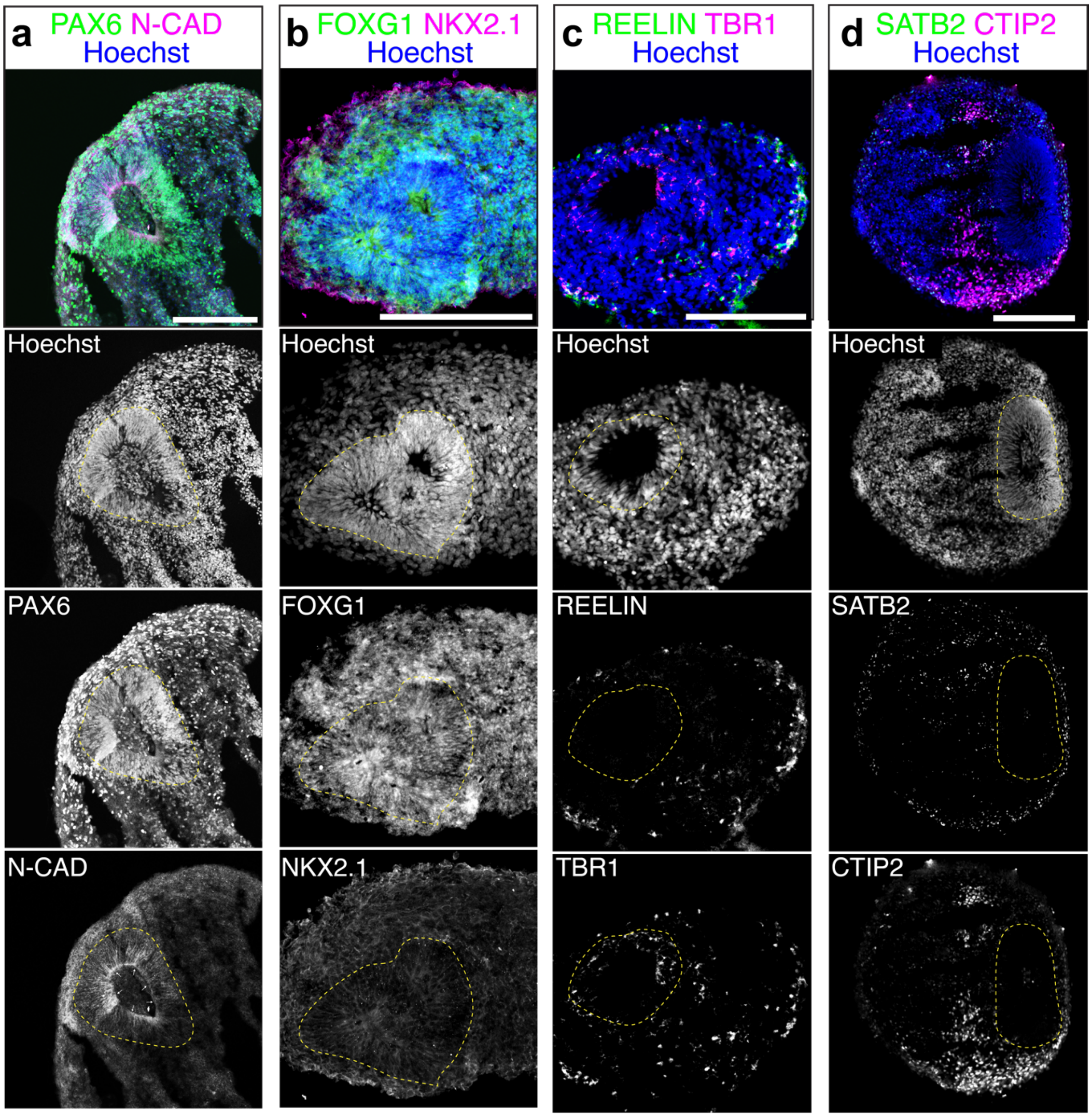

Supplementary Figure 6. Images of organoid sections immunostained with antibodies against different cell-type specific markers.

a, PAX6 and N-Cad. **b**, FOXG1 and NKX2.1. **c**, REELIN and TBR1. **d**, SATB2 and CTIP2. Scale bars = 200 μm.

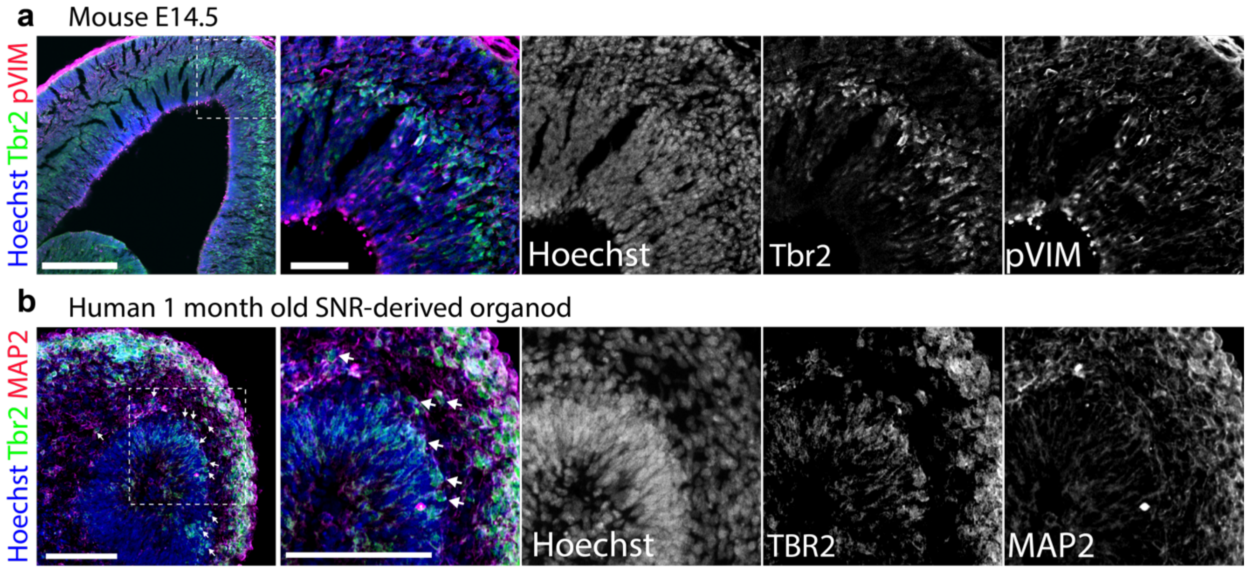

Supplementary Figure 7. Images of mouse and human 1-month-old organoid sections immunostained with anti-TBR2 antibodies.

a, Embryonic (E14.5) mouse brain section immunostained with antibodies against Tbr2 and pVIM were used as positive control to validate anti-Tbr2 antibody. **b**, Image of 1-month-old organoid section immunostained with anti-TBR2 and MAP2 antibodies. Scale bars = 100 and 50 μm.

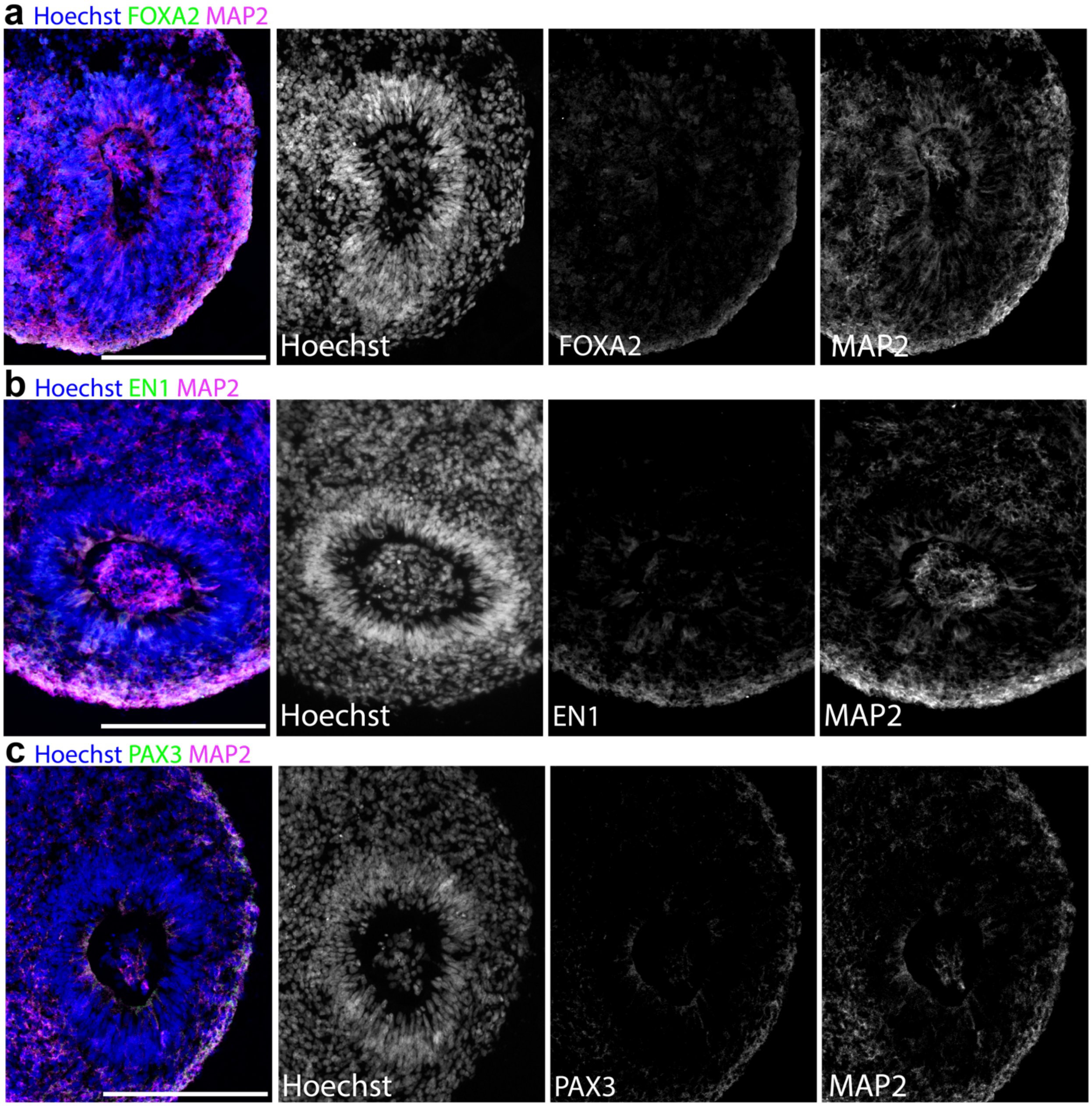

Supplementary Figure 8. Images of organoid sections immunostained with antibodies against posterior cell-type specific markers.

a, FOXA2 and MAP2; **b**, EN1 and MAP2; and **c**, PAX3 and MAP2. Scale bars = 50 μm.

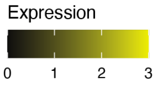

Differentially expressed genes in defferent clusters (1-month-old organoids)

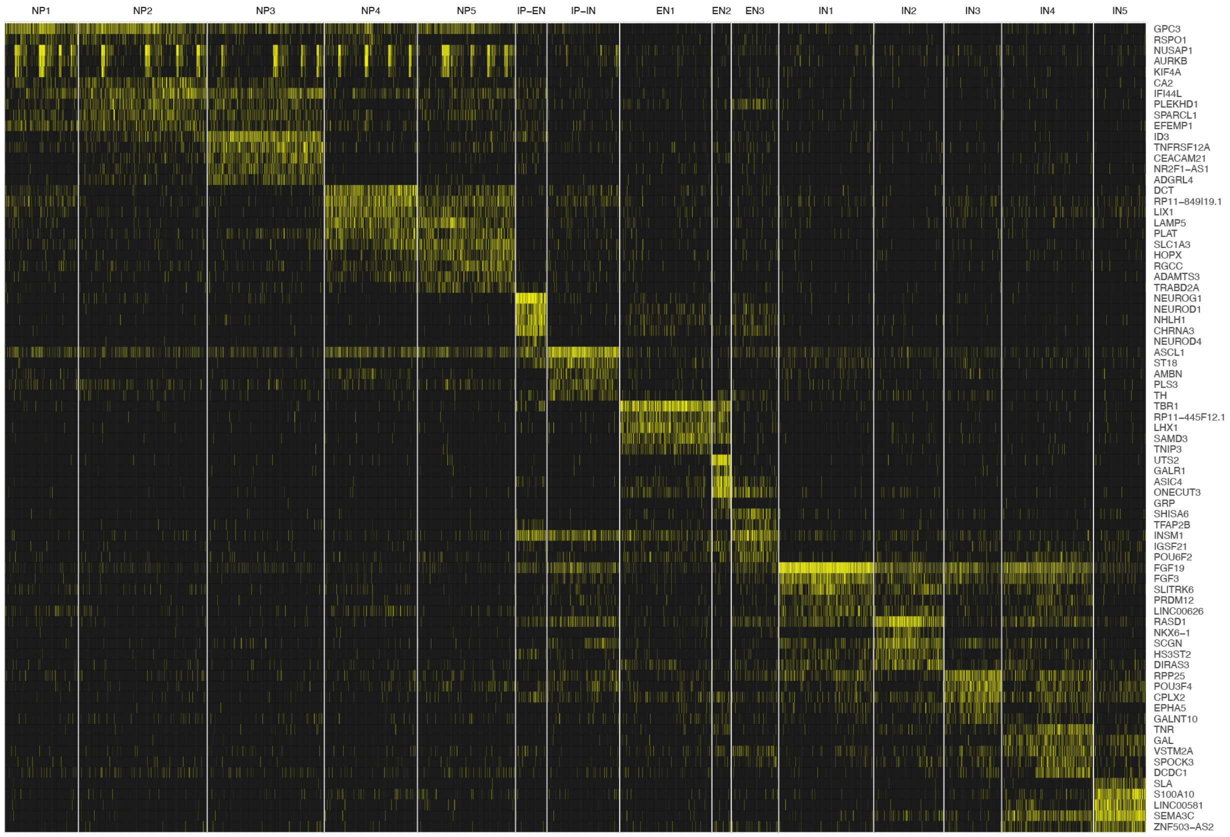

Differentially expressed genes in defferent clusters (5-month-old organoids)

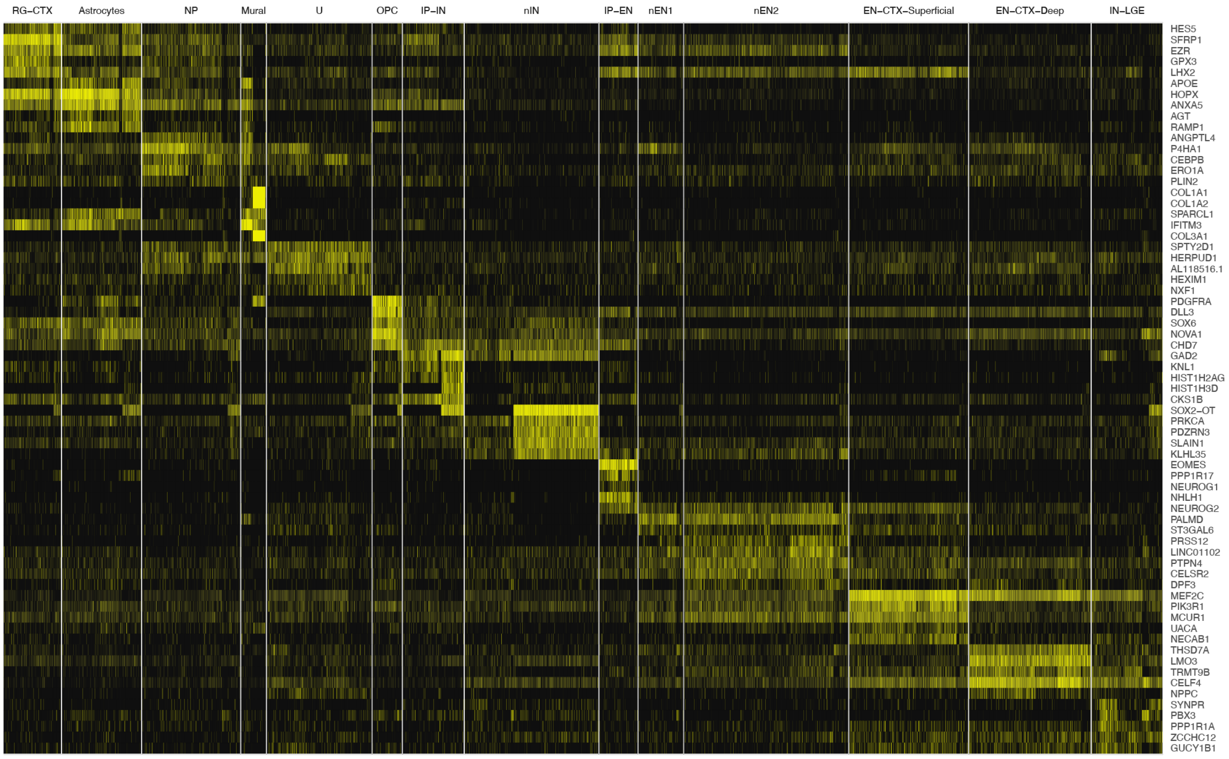

Supplementary Figure 9. Cell cluster-specific marker genes in SNR-derived organoids

Heatmap visualization of the 5 most significantly enriched genes for each cluster in 1- (top) and 5-month-old organoids (bottom). Additional information regarding the gene expression profiles of cells in different clusters can be found in Table SX or at http://organoid.chpc.utah.edu:4242

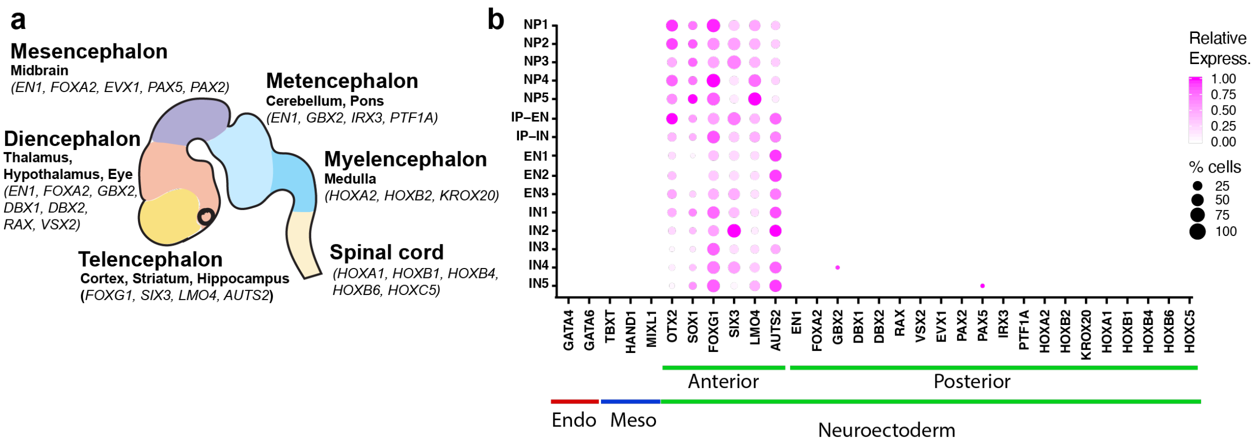

Supplementary Figure 10. Expression of typical endodermal, mesodermal, and neuroectodermal markers in 1-month-old SNR-derived organoids

a, Cartoon depicting embryonic brain and region-specific markers. **b,** Dot-plot visualization of region- and cell-type specific marker expression in different cell clusters.

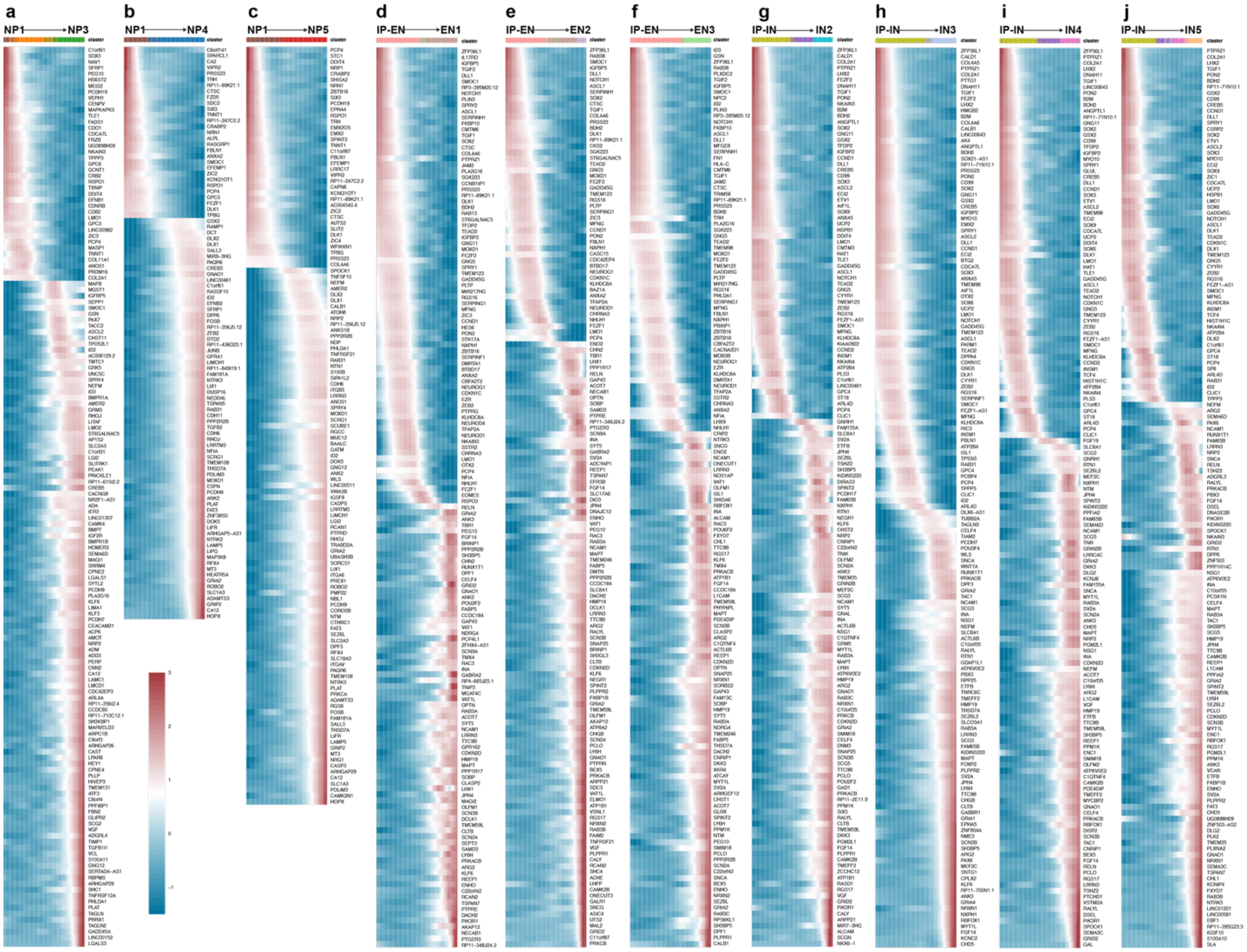

Supplementary Figure 11. Trajectory-specific gene

a-j, Smoothed gene expression heat maps of the top genes differentially expressed along NP1 -> NP3 (**a**), NP1 -> NP4 (**b**), NP1 -> NP5 (**c**), IP-EN -> EN1 (**d**), IP-EN -> EN2 (**e**), IP-EN -> EN3 (**f**), IP-IN -> IN2 (**g**), IP-IN -> IN3 (**h**), IP-IN -> IN4 (**i**), and IP-IN -> IN5 (**j**) trajectories. Genes showing differential expression displayed vertically. Horizontal axes display pseudotime. The genes are ordered by peak expression time on the pseudotime axis.

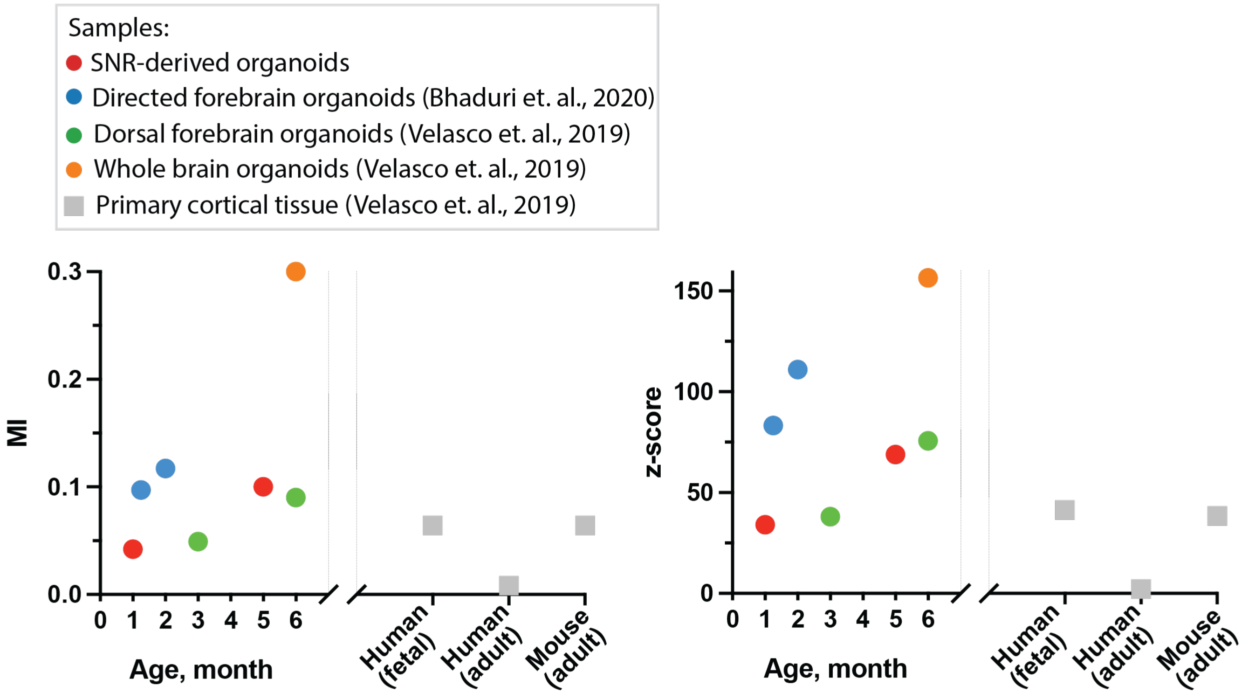

Supplementary Figure 12. Characterization of reproducibility in organoids and primary brain tissue.

Mutual information (MI) between cluster assignment and sample identity was computed to assess the reproducibility in organoids and brain tissue. A lower MI and z-score (the divergence of MI score from the score expected at random) indicates higher reproducibility. The plots compare the MI (left) and z-score (right) of SNR-derived organoids against organoids generated by other differentiation protocols (Velasco et al., 2019 and Bhaduri et al., 2020)[2,3] across different developmental stages. The scores for organoids from our study and Bhaduri et al., 2020 are computed following the approach described in Velasco et al., 2019. Source data are provided as a Source Data file.

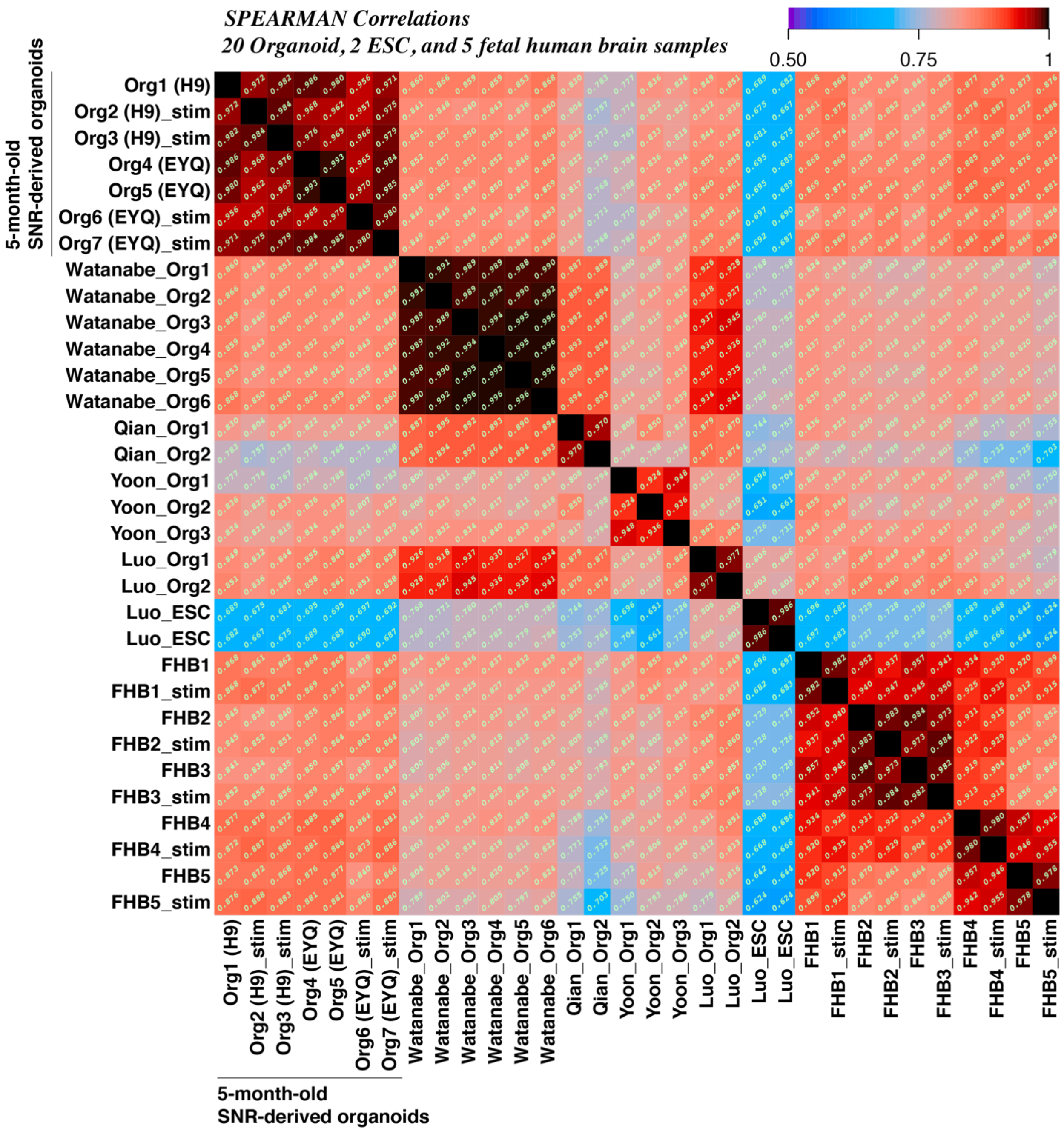

Supplementary Figure 13. Transcriptional analysis to assess reproducibility across multiple studies.

Spearman correlations of expression profiles among 20 organoids ("Org"), 2 embryonic stem cell samples ("ESC"), and 10 fetal human brain ("FHB") samples. Pairwise correlation values shown

by color scale and displayed in each off-diagonal cell. The following samples were used for analysis:

5-month-old SNR-derived telencephalic organoids: 7 biological replicates produced from H9 and EYQ stem cell lines, unstimulated or stimulated ("stim") with KCl for 6 hours;

2-month-old cortical organoids (Watanabe et al., Cell Reports 2017)[4]: 6 biological replicates produced from H9 stem cell line (GEO accession numbers: GSM2580319 [Org1], GSM2580321 [Org2], GSM2580323 [Org3], GSM2580325 [Org4], GSM2580327 [Org5], GSM2580329 [Org6]);

3.5-month-old telencephalic organoids (Qian et al., Cell 2016)[5]: 2 biological replicates produced from an iPSC stem cell line (GEO accession numbers: GSM2112671 [Org1] and GSM2112672 [Org2]);

3.5-month-old cortical spheroids (Yoon et al., Nature Methods 2019)[6]: 3 biological replicates produced from three different iPSC stem cell lines (GEO accession numbers: GSM3408648 [Org1], GSM3408667 [Org2], and GSM3408685 [Org3]);

2-month-old cerebral organoids (Luo et al., Cell Reports 2016)[7]: 2 biological replicates produced from H9 stem cell line (GEO accession numbers: GSM2180144 [Org1] and GSM2180145 [Org2]);

H9 embryonic stem cells (Luo et al., Cell Reports 2016)[7]: 2 biological replicates (GEO accession numbers: GSM2180138 [ESC1] and GSM2180139 [ESC2]);

Fetal human brain (FHB) samples (Ataman et al., Nature 2016)[8]: 5 biological replicates, unstimulated or stimulated ("stim") with KCl for 6 hours (GEO accession numbers:

GSM2072621, GSM2072624, GSM2072627, GSM2072630, GSM2072633, GSM2072623, GSM2072626, GSM2072629, GSM2072632, and GSM2072635).

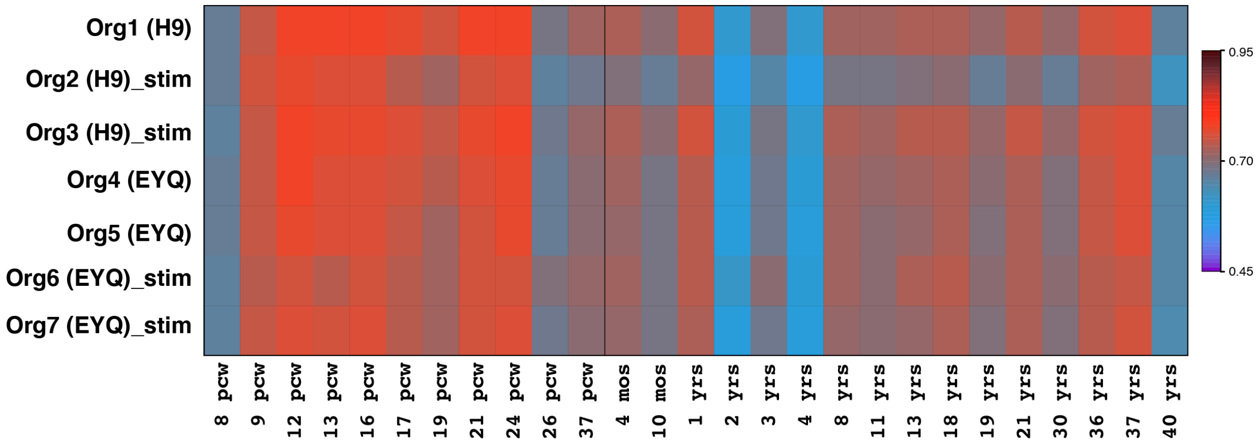

Supplementary Figure 14. Transcriptional analysis to assess the developmental age of 5-month-old SNR-derived organoids.

Spearman correlation of expression levels between 7 individual 5-month-old SNR-derived organoids and age-specific neocortical human samples obtained from BrainSpan database (http://www.brainspan.org/).

The following neocortical samples from BrainSpan samples were used for analysis: VFC (38 samples at 27 ages), MFC (37 samples at 28 ages), DFC (39 samples at 29 ages). Horizontal axis displays age on a quasi-log scale, beginning at zero post-conception weeks (pcw), through birth (at 40 pcw), up to 50 years; range of ages plotted, 8 pcw to 40 years old.

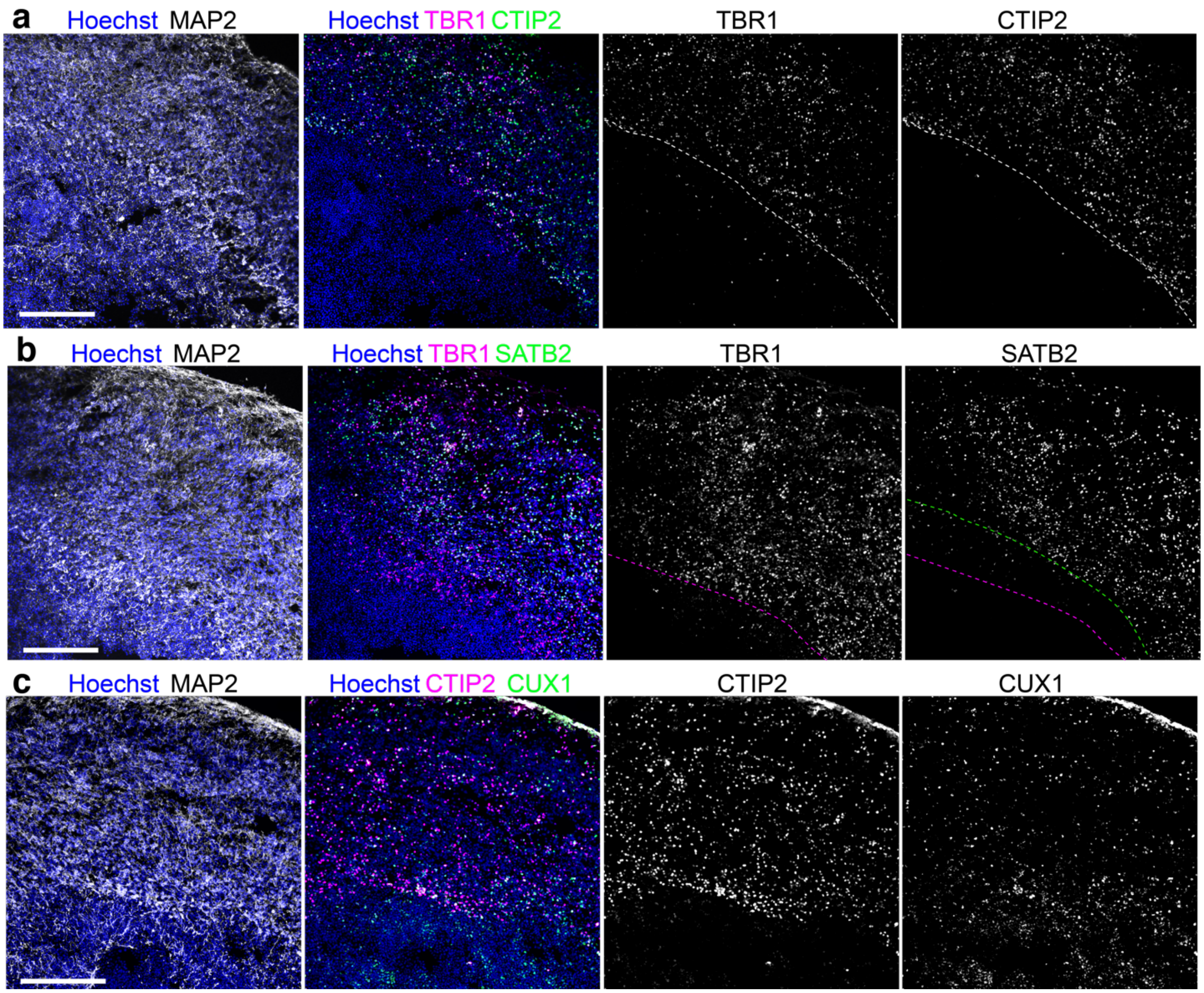

Supplementary Figure 15. Images of sections obtained from 5-month-old organoids immunostained with different cortical layer markers.

a-c, Organoid sections were obtained from 2242-5 stem cell line and immunostainied with antibodies against MAP2, TBR1 and CTIP2 (**a**), MAP2, TBR1 and SATB2 (**b**), and MAP2, CTIP2 and CUX1 (**c**). Cropped images are presented in Fig. 7b-c. Scale bars = 200 µm.

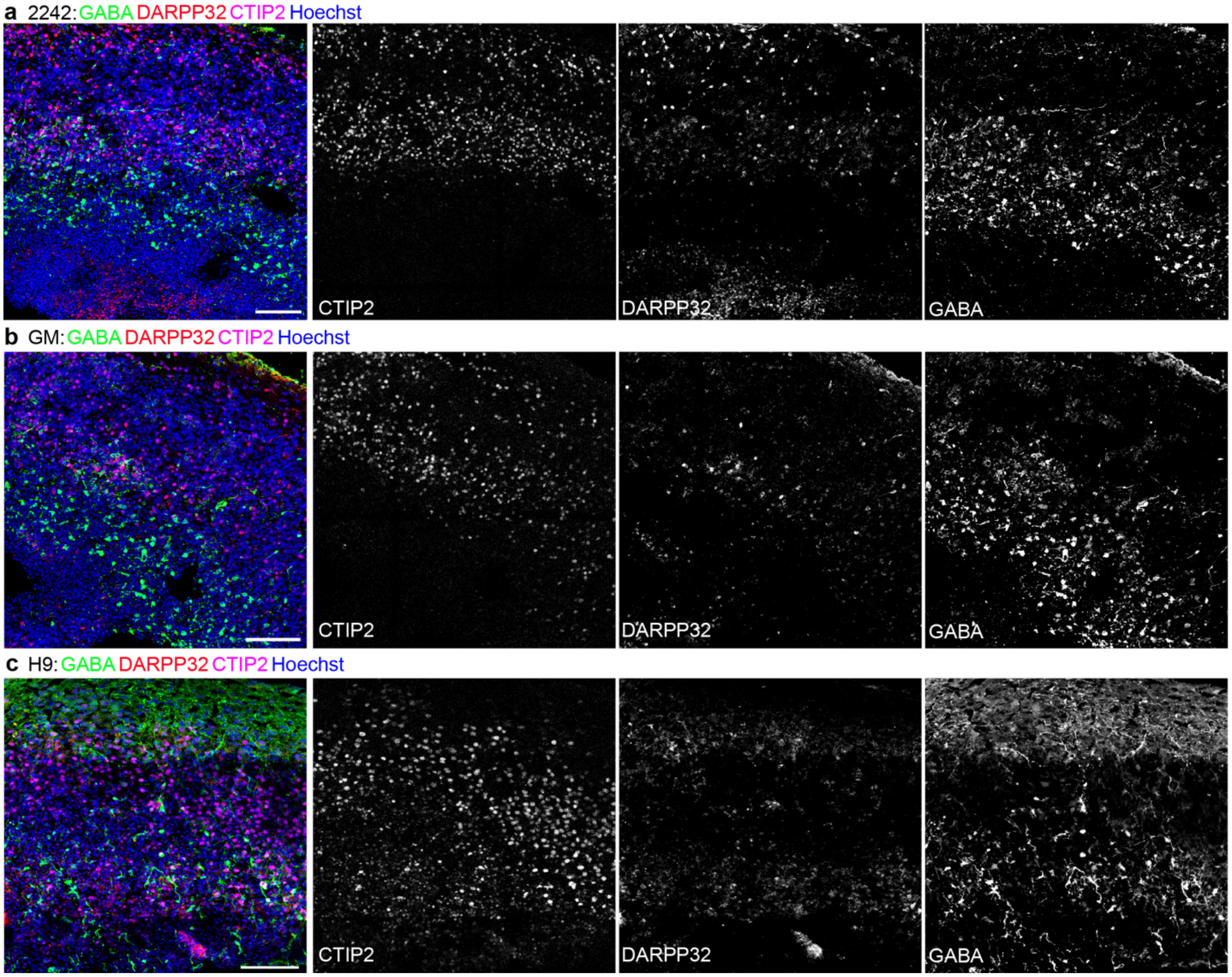

Supplementary Figure 16. Images of sections obtained from 5-month-old organoids immunostained with cortico-striatal cell-type specific markers.

a-c, Organoid sections were obtained from 2242-5 (**a**), GM07492 (**b**), and H9 (**b**) stem cell lines and immunostainied with antibodies against GABA, DARPP32, and CTIP2. Scale bars = 200 μm.

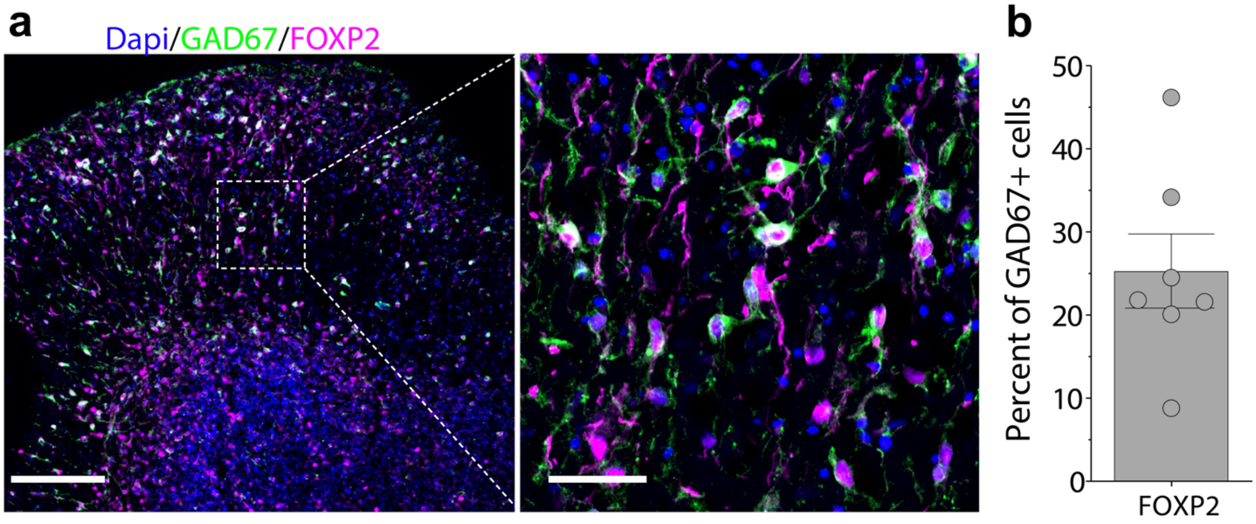

Supplementary Figure 17. Identification and characterization of inhibitory striatal projection neurons in 5-month-old SNR-derived organoids.

a, Organoid sections immunostained for GAD67 and FOXP2. **b**, Percentage of inhibitory neurons (GAD67-expressing cells) that express FOXP2 (n = 7 organoids/3–10 sections per organoid in produced 1-3 batches from H9, 2242-5, and EP2-15 lines). Data are presented as means ± s.e.m. Scale bars = 200 and 50 µm. Source data are provided as a Source Data file.

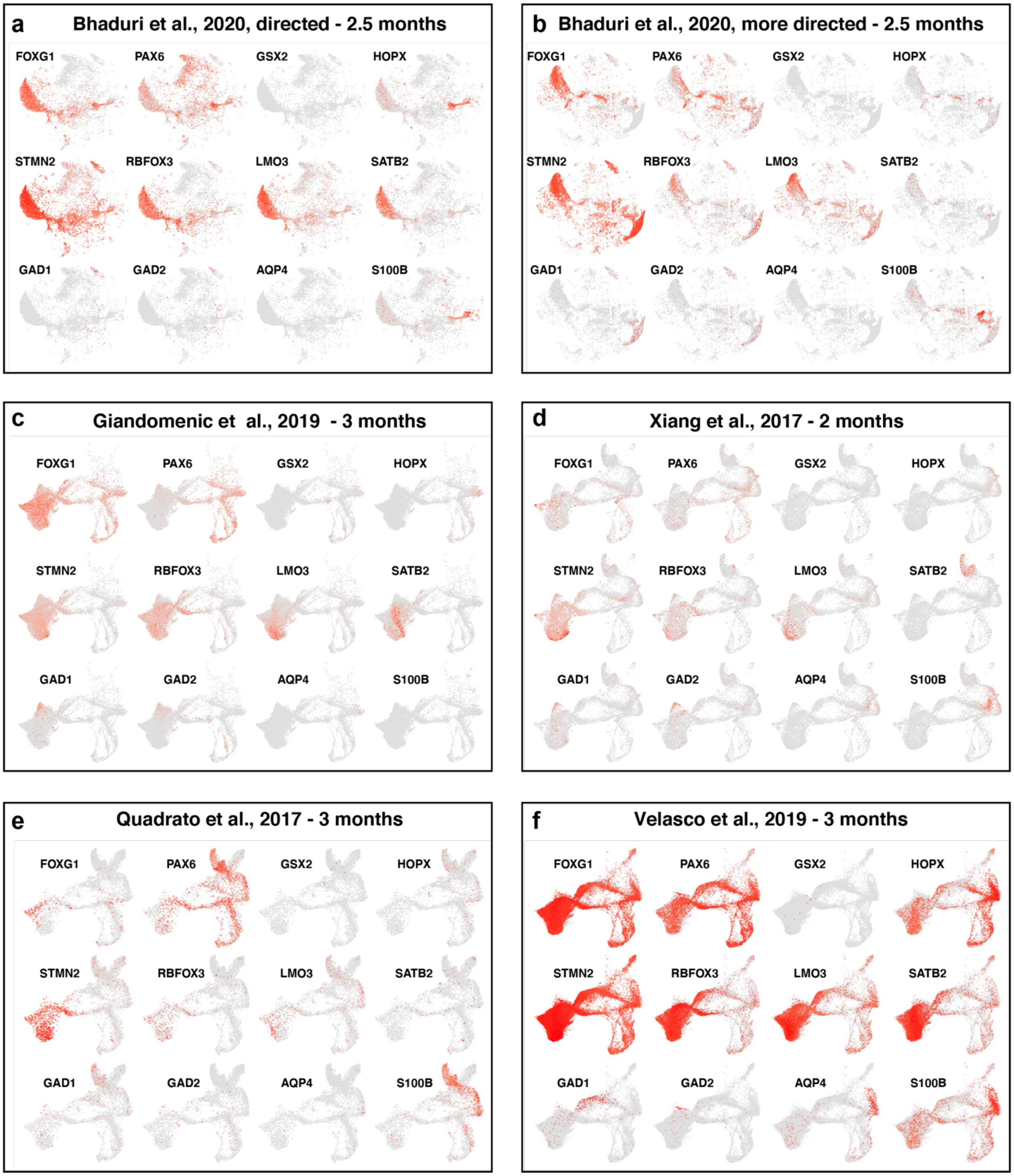
a
Bhaduri et al., 2020, directed - 2.5 months
FOXG1
PAX6
GSX2
HOPX
STMN2
RBFOX3
LMO3
SATB2
GAD1
GAD2
AQP4
S100B
b
Bhaduri et al., 2020, more directed - 2.5 months
FOXG1
PAX6
GSX2
HOPX
STMN2
RBFOX3
LMO3
SATB2
GAD1
GAD2
AQP4
S100B
c
Giandomenic et al., 2019 - 3 months
FOXG1
PAX6
GSX2
HOPX
STMN2
RBFOX3
LMO3
SATB2
GAD1
GAD2
AQP4
S100B
d
Xiang et al., 2017 - 2 months
FOXG1
PAX6
GSX2
HOPX
STMN2
RBFOX3
LMO3
SATB2
GAD1
GAD2
AQP4
S100B
e
Quadrato et al., 2017 - 3 months
FOXG1
PAX6
GSX2
HOPX
STMN2
RBFOX3
LMO3
SATB2
GAD1
GAD2
AQP4
S100B
f
Velasco et al., 2019 - 3 months
FOXG1
PAX6
GSX2
HOPX
STMN2
RBFOX3
LMO3
SATB2
GAD1
GAD2
AQP4
S100B

Supplementary Figure 18. Expression of selected cell-specific markers in telencephalic organoids.

a-f, Heat map visualizations of FOXG1, PAX6, HOPX, GSX2, STMN, RBFOX3 (NeuN), LMO3, SATB2, GAD1/2, and AQP4, and S100B expression in telencephalic organoids reported by Bhaduri et. al., 2020[3] (**a-b**), Giandomenico et al., 2019[9] (**c**), Xiang et al., 2017[10] (**d**), Quadrato et al., 2017[11] (**e**), and Velasco et al., 2019[2] (**f**). The single-cell RNAseq datasets were downloaded from the USCS Cell Browser (https://organoidreportcard.cells.ucsc.edu for Bhaduri et al., 2020[3] and https://organoidatlas.cells.ucsc.edu for other datasets preprocessed by Tanaka et al. 2020[12]). These datasets contain the gene expression profile of each cell and pre-computed t-SNE/UMAP embeddings.

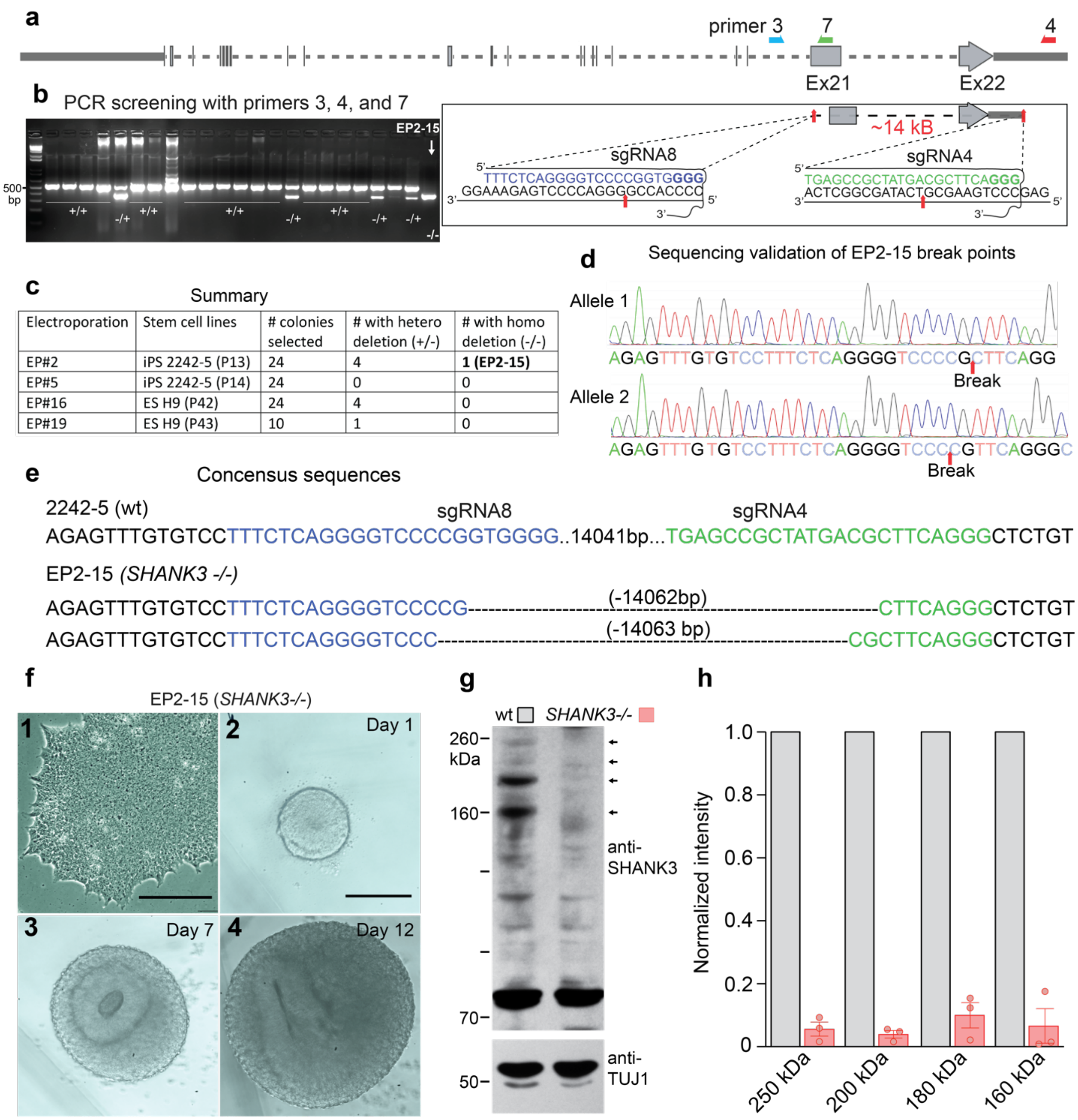

Supplementary Figure 19. Generation and characterization of CRISPR/Cas9-engineered *SHANK3-/-* iPSC line.

a, Strategy used for introducing *SHANK3* deletion into human iPSCs. Two single-guide RNAs (sgRNA), sgRNA8 and 4, were designed to flank exons 21-22 and to introduce a ~14-kB deletion (zoom in). **b**, PCR verification of the deletion using a set of primers that recognize

different regions in the proximity of the exons 21-22. **c**, Homozygous deletion of *SHANK3* was detected in 1 out of 82 tested colonies (EP2-15 clone). **d**, Verification of homozygous *SHANK3* deletion in EP2-15 line using subcloning and sequencing. **e**, Consensus sequences of exons 21-22 region in 2242 (isogenic control) and EP2-15 (*SHANK3-/-*). **f**, Images of iPSCs (**f1**), iPSC-derived neural rosette (**f2**), and SRCOs at different time points after single rosette isolation (**f3-4**). **g**, Images of Western blots of lysates obtained from wild-type (WT) and *SHANK3-/-* neurons immunoblotted using anti-SHANK3 and anti-TUJ1 antibodies. **h**, Quantification of expression of different isoforms of SHANK3 in wt and *SHANK3-/-* neurons (n = 3 pairs of samples). Data are presented as means ± s.e.m. Full scan blots are provided in the source data file. Scale bars = 250 μm. Source data are provided as a Source Data file.

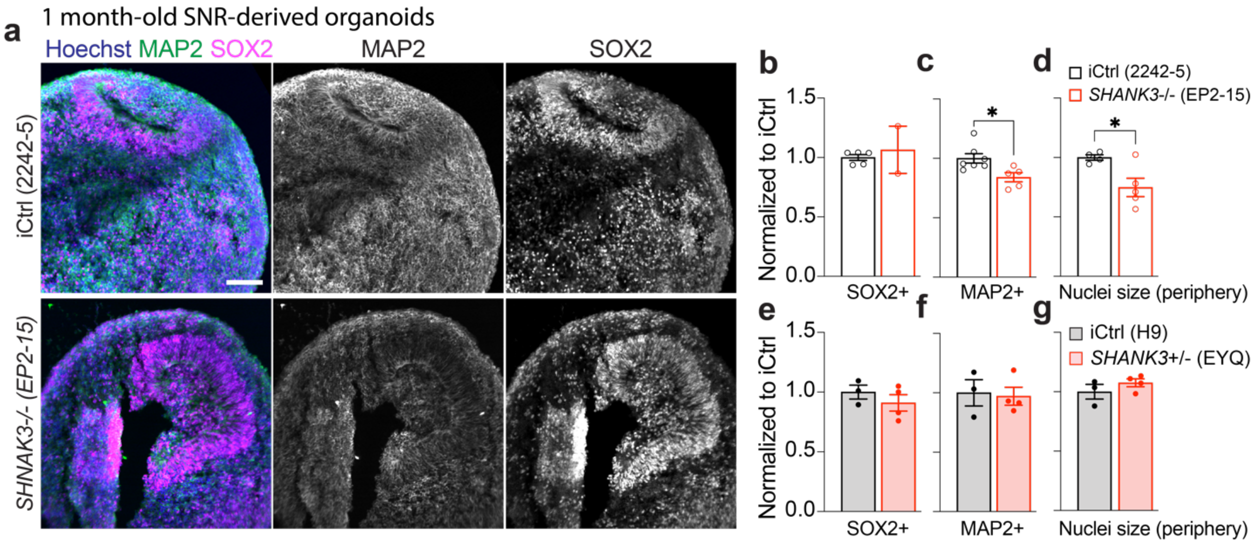

Supplementary Figure 20. Characterization of size and neurogenesis deficits in SHANK3-deficient organoids.

a, Images of 1-month-old organoid sections from iCtrl (2224-5) and *SHANK3*-/- (EP2-15) lines stained with anti-SOX2 and MAP2 antibodies and Hoechst. **b-g**, Quantifications of proportions of SOX2- and MAP2-expressing cells in *SHANK3*-/- (**b-d**) and *SHANK3*+/- (**e-g**) organoids as compared to respective iCtrl organoids (SOX2, n = 5 iCtrl [2224-5], 2 *SHANK3*-/- [EP2-15], 3 iCtrl [H9], and 4 *SHANK3*+/- [EYQ2-20] organoids; MAP2, n = 7 iCtrl [2224-5], 5 *SHANK3*-/- [EP2-15], 3 iCtrl [H9], and 4 *SHANK3*+/- [EYQ2-20] organoids; nuclei size, n = 4 iCtrl [2224-5], 5 *SHANK3*-/- [EP2-15], 3 iCtrl [H9], and 4 *SHANK3*+/- [EYQ2-20] organoids). The combined quantifications are also presented in Fig. 1j. Data presented as mean ± s.e.m.; *P<0.05, unpaired two-sided t-test. Scale bars = 50 (**a, e**) and 5 µm (**e**). Source data are provided as a Source Data file.

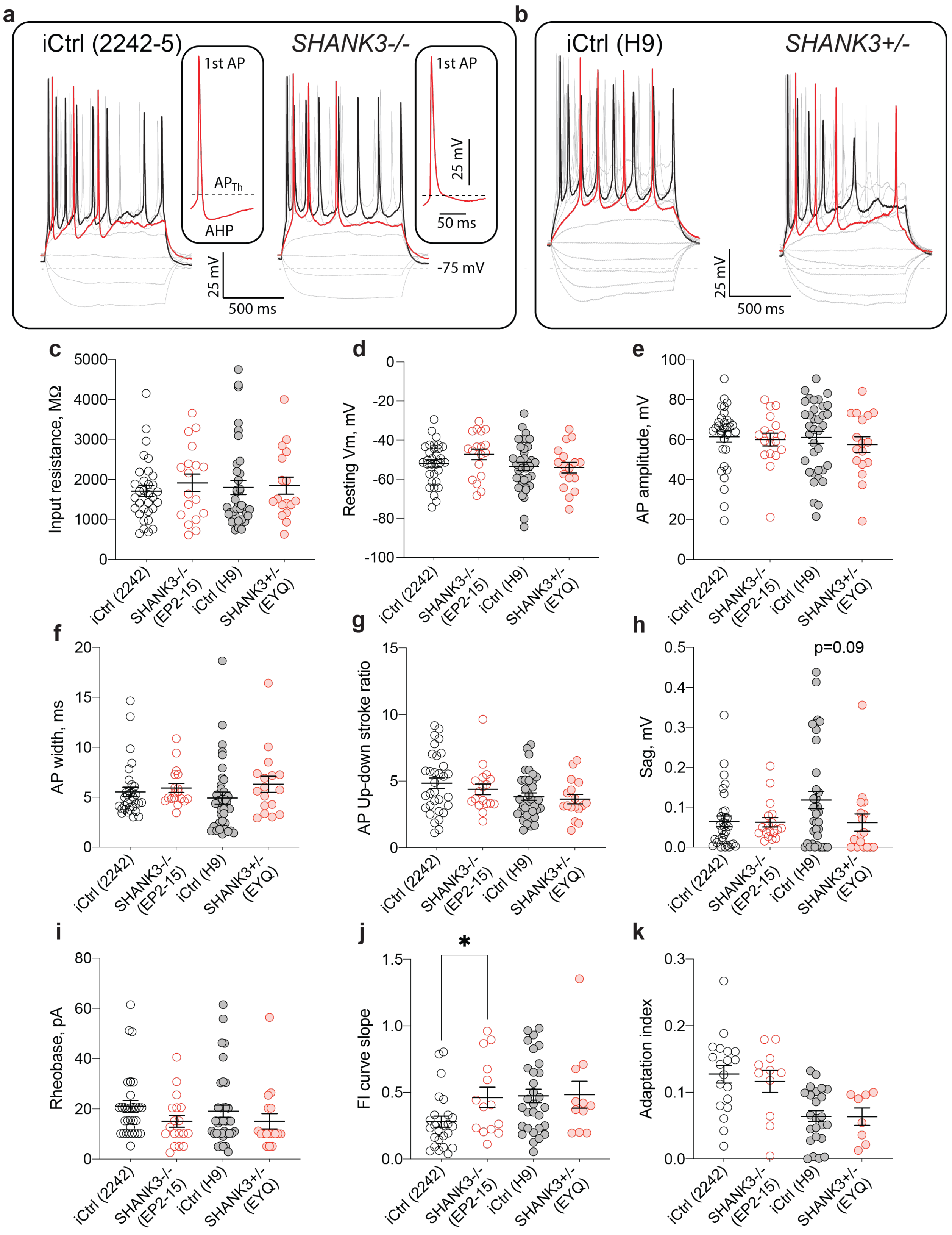

a
iCtrl (2242-5)
1st AP
AP_Th
AHP
SHANK3-/-
1st AP
25 mV
50 ms
-75 mV
25 mV
500 ms
b
iCtrl (H9)
SHANK3+/-
25 mV
500 ms
c
Input resistance, MΩ
d
Resting Vm, mV
e
AP amplitude, mV
f
AP width, ms
g
AP Up-down stroke ratio
h
Sag, mV
p=0.09
i
Rheobase, pA
j
FI curve slope
*
k
Adaptation index
iCtrl (2242)
SHANK3-/- (EP2-15)
iCtrl (H9)
SHANK3+/- (EYQ)

Supplementary Figure 21. Characterization of intrinsic excitability properties of neurons in iCtrl and SHANK3-deficient organoids.

a-b, Traces of membrane potentials obtained from SHANK3-/- (**a**) and SHANK3+/- (**b**) neurons as compared to respective isogenic control (iCtrl) neurons in response to different somatic current injections. **c-k**, Quantification of input resistance (**c**), resting membrane potential (**d**), amplitude of the first AP (**e**), width of the first AP (**f**), ratio of the first AP up to down stroke (**g**), sag amplitude (**h**), rheobase (**i**), slope of frequency-current curve (**j**), and adaptation index (**k**). (n = 32, 18, 36, and 17 cells, from 4 iCtrl [2242-5], 3 *SHANK3-/-* [EP2-15], 11 iCtrl [H9], and 2 *SHANK3+/-* [EYQ] organoids). *P=0.029, unpaired two-sided t-test. These cells are also included in analyses presented in Figs. 8g and 9d. Data presented as individual data points and mean ± s.e.m. Source data are provided as a Source Data file.

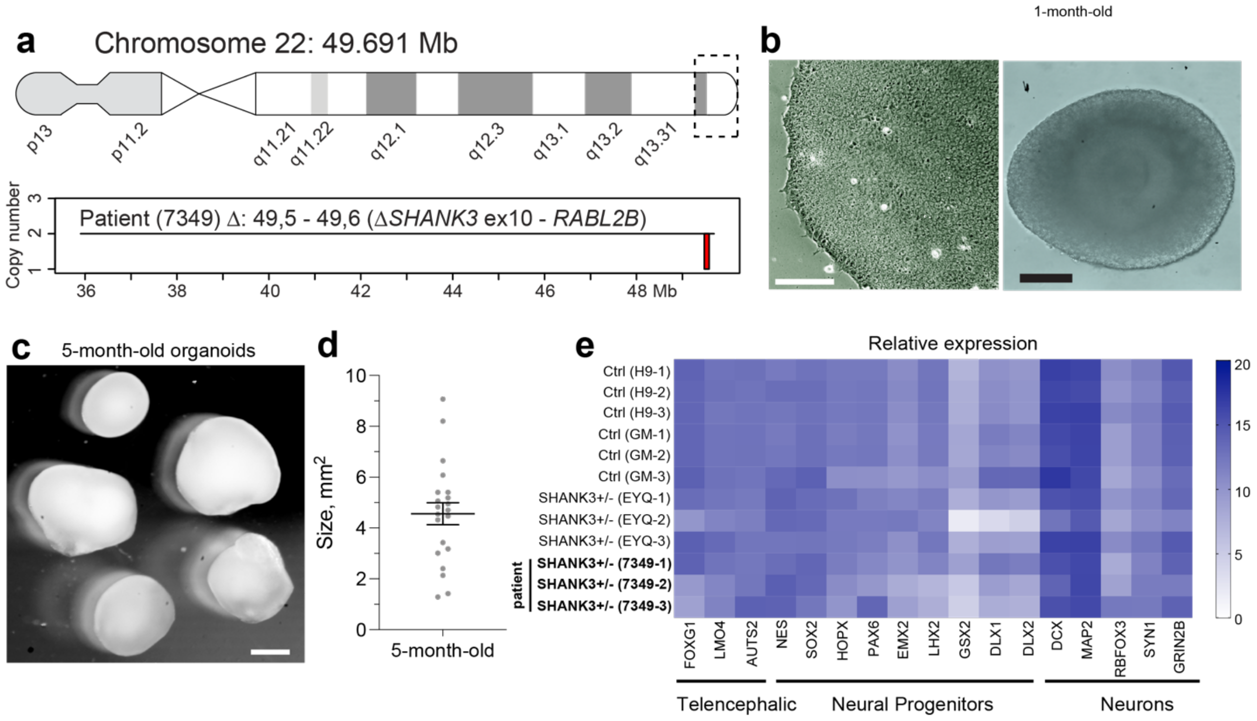

Supplementary Figure 22. Characterization of SNR-derived organoids generated from *SHANK3*+/- patient iPSC line.

a, Identification of genetic abnormality in patient fibroblasts using SNP array. **b,** Images of patient iPSC colony and early SNR-derived organoids. **c,** Images of patient 5-month-old SNR-derived organoids. **d**, Quantification of 5-month-old organoid sizes (n = 21 organoids produced in 3 differentiation batches). **e,** Heatmap visualization of normalized expression of cell type-specific marker genes in 5-month-old control and *SHANK3*+/- organoids. Data presented as mean (centre) ± s.e.m. (error bars); Scale bars = 200 (**b**) and 1000 μm (**c**). Source data are provided as a Source Data file.

Supplementary Table 1: List of human stem cell lines and organoids produced from these lines that were used in different experiments.

Line	Source	Genotype	Sex	Number of independent differentiation batches	Number of organoids used in different experiments					
					scRNA-seq		bulk RNA-seq	Electrophysilogy	Immunohistochemistry	
					1 month	5 month	5 month	5 month	1 month	5 month
2242-5	iPSC line, Dolmetsch lab, Stanford	normal	XY	5	2	1		4	7	3
EP2-15	iPSC line, generated from 2224-5, Sheglovitov lab, University of Utah	*SHANK3* -/- (Δex21-22)	XY	4				4	5	4
H9	ESC line, WiCell	normal	XX	8	2	2	3	10	3	3
EYQ2-20	ESC line, generated from H9, Sheglovitov lab, University of Utah	*SHANK3* +/- (Δex1-22)	XX	7		2	3	2	4	3
GM07492	iPSC line, Ernst lab, McGill University	normal	XY	1		1	3			
7349-3	iPSC line, generated from patient fibroblasts, Shcheglovitov lab, University of Utah	*SHANK3* +/- (Δex10-*RABL2B*)	XY	3		1	3			

Supplementary Table 2: List of primary and secondary antibodies used in this study.

Antibody target	Company	Catalog #	Raised in	Dilution
Sox2	Abcam	ab97959	Rb	1:500
Pax6	BioLegend/Covance	901301 (previously PRB-278P)	Rb	1:250
Gsx2	Thermo Fisher	PA5-35887	Rb	1:250
N-Cadherin	Abcam	ab98952	Ms	1:500
Ki67	BD Biosciences	550609	Ms	1:100
Tuj1	Covance	MMS-435P	Ms	1:1000
Map2	Synaptic Systems	188 004	Gp	1:1000
Tbr2	EMD Millipore	AB2283	Rb	1:300
Reelin	MBL International	D223-3	Ms	1:500
GABA	Sigma	A2052	Rb	1:500
GAD67	EMD Millipore	MAB5406	Ms	1:500
Somatostatin	Chemicon	AB5494	Rt	1:500
Parvalbumin	Swant	PV 235	Ms	1:500
VIP	Immunostar	20077	Rb	1:500
Calretinin	Swant	7697	Rb	1:500
Calbindin (d28k)	Swant	300	Ms	1:500
Darpp32	Santa Cruz	sc-271111	MS	
S100b	Agilent Technologies	Z031129-2	Rb	1:1000
GFAP	Abcam	ab4674	Ch	1:300
MBP	Chemicon	MAB386	Rt	1:500
O4	R&D Systems	MAB1326-SP	Ms	1:500
Tbr1	Abcam	ab31940	Rb	1:500
Ctip2	Abcam	ab18465	Rt	1:500
Satb2	Abcam	ab51502	Ms	1:500
Cux1	Santa Cruz Biotechnology	sc-13024	Rb	1:500
GFP	Abcam	ab13970	Ch	1:1000
Caspase-3	BD Pharmingen	559565	Rb	1:500
PH3	EMD Millipore	06-570	Rb	1:500
Foxp2	Abcam	ab16046	Rb	1:500
Bassoon	Enzo Life Sciences	ADI-VAM-PS003-D	Ms	1:500
Homer1	Synaptic Systems	160 004	Gp	1:500
Gephyrin	Synaptic Systems	147 003	Rb	1:50
Vglut1	EMD Millipore	AB5905	Gp	1:1000
Synapsin1	Synaptic Systems	106 001	Rb	1:500
PSD-95	Abcam	ab2723	Ms	1:100
Shank1	Novus Biologicals	NB300-167	Rb	1:100

Shank2	Synaptic Systems	162 202	Rb	1:200
Shank3	Synaptic Systems	162 304	Gp	1:200
Shank3	Synaptic Systems	162 302	Rb	1:100
PDGFR-β	Santa Cruz Biotechnology	sc-374573	Ms	1:100
FoxG1 (Bf1)	Takara Bio	M227	Ms	1:250
Fam107A	Proteintech	12176-1-AP	Rb	1:100
αSMA	Thermo Fisher Scientific	710487	Rb	1:100
Alexa Fluor 405 goat anti-mouse IgG (H+L)	Thermo Fisher Scientific	A31553		1:500
Alexa Fluor 488 goat anti-mouse IgG (H+L)	Thermo Fisher Scientific	A11029		1:500
Alexa Fluor 488 goat anti-mouse IgM (μ chain)	Thermo Fisher Scientific	A21042		1:500
Alexa Fluor 488 goat anti-rabbit IgG (H+L)	Thermo Fisher Scientific	A11034		1:500
Alexa Fluor 488 goat anti-rat IgM (μ chain)	Thermo Fisher Scientific	A21212		1:500
Alexa Fluor 488 Donkey anti Sheep IgG (H+L)	Thermo Fisher Scientific	A11015		1:500
Alexa Fluor 568 goat anti-Rabbit IgG (H+L)	Thermo Fisher Scientific	A11036		1:500
Alexa Fluor 594 goat anti-guinea pig IgG (H+L)	Thermo Fisher Scientific	A11076		1:500
Alexa Fluor 594 goat anti-mouse IgG (H+L)	Thermo Fisher Scientific	A11005		1:500
Alexa Fluor 594 goat anti-rat IgM (μ chain)	Thermo Fisher Scientific	A21213		1:500
Alexa Fluor 647 goat anti-mouse IgG (H+L)	Thermo Fisher Scientific	A21235		1:500
Alexa Fluor 647 goat anti-guinea pig IgG (H+L)	Thermo Fisher Scientific	A21450		1:500
Alexa Fluor 647 goat anti-Rabbit IgG (H+L)	Thermo Fisher Scientific	A21245		1:500

Alexa Fluor 647 goat anti-Rat IgG (H+L)	Thermo Fisher Scientific	A21247		1:500
Streptavidin, Alexa Fluor 647 conjugate	Thermo Fisher Scientific	S21374		1:500

Abbreviations: Rb –rabbit, Ms – mouse, Gp – guinea pig, Rt – rat, Ch –chicken

Supplementary References:

1. Xie, Y. *et al.* Lef1-dependent hypothalamic neurogenesis inhibits anxiety. *PLoS Biol.* **15**, 1–27 (2017).
2. Velasco, S. *et al.* Individual brain organoids reproducibly form cell diversity of the human cerebral cortex. *Nature* **570**, 523–527 (2019).
3. Bhaduri, A. *et al.* Cell stress in cortical organoids impairs molecular subtype specification. *Nature* **578**, 142–8 (2020).
4. Watanabe, M. *et al.* Self-Organized Cerebral Organoids with Human-Specific Features Predict Effective Drugs to Combat Zika Virus Infection. *Cell Rep.* **21**, 517–532 (2017).
5. Qian, X. *et al.* Brain-Region-Specific Organoids Using Mini-bioreactors for Modeling ZIKV Exposure. *Cell* **165**, 1238–1254 (2016).
6. Yoon, S. J. *et al.* Reliability of human cortical organoid generation. *Nat. Methods* **16**, 75–78 (2019).
7. Luo, C. *et al.* Cerebral Organoids Recapitulate Epigenomic Signatures of the Human Fetal Brain. *Cell Rep.* **17**, 3369–3384 (2016).
8. Ataman, B. *et al.* Evolution of Osteocrin as an activity-regulated factor in the primate brain. *Nature* **539**, 242–247 (2016).
9. Giandomenico, S. L. *et al.* Cerebral organoids at the air–liquid interface generate diverse nerve tracts with functional output. *Nat. Neurosci.* **22**, 669–679 (2019).
10. Xiang, Y. *et al.* Fusion of Regionally Specified hPSC-Derived Organoids Models Human Brain Development and Interneuron Migration. *Cell Stem Cell* **21**, 383-398.e7 (2017).
11. Quadrato, G. *et al.* Cell diversity and network dynamics in photosensitive human brain organoids. *Nature* **545**, 48–53 (2017).
12. Tanaka, Y., Cakir, B., Xiang, Y., Sullivan, G. J. & Park, I. H. Synthetic Analyses of Single-Cell Transcriptomes from Multiple Brain Organoids and Fetal Brain. *Cell Rep.* **30**, 1682-1689.e3 (2020).
