# Supplementary material for: Modeling human telencephalic development and autism-associated SHANK3 deficiency using organoids generated from single neural rosettes
Source: Nat Commun. 2022 Oct 6;13:5688. doi: 10.1038/s41467-022-33364-z (PMC9537523; doi:10.1038/s41467-022-33364-z)
Supplement: Supplementary file 3 — Description of Additional Supplementary Files [file 41467_2022_33364_MOESM3_ESM.pdf]

Description of Additional Supplementary Files

File Name: Supplementary Data 1
Description: List of all cluster-specific genes detected in different clusters in 1-month-old SNR-derived organoids. Abbreviations: avg_logFC - Log fold-change of the average gene expression; pct.1 - the percentage of cells with detected gene expression in the target cluster; pct.2 - the percentage of cells with detected gene expression in the background cluster; p_val – unadjusted p-value; p_val_adj - p-value adjusted using the Bonferroni multiple comparison test.

File Name: Supplementary Data 2
Description: List of all trajectory-specific genes detected expressed along different trajectories in 1-month-old SNR-derived organoids. Abbreviations: celltype – name of cell type; curve – name of trajectory curve; start – trajectory beginning cluster; end - trajectory end cluster; gene – gene name; pval - unadjusted p-value; pval_adj – p-value adjusted using the Benjamini-Hochberg multiple comparison test; pct_exp - percentage of cells along the trajectory that express the gene; fold_change - fold change of gene expression from the minimum expression to the peak; peak –pseudotime at which the peak expression was detected; stage - early or late stage relative to the peak.

File Name: Supplementary Data 3
Description: List of gene ontology (GO) terms enriched in association with different trajectories in 1-month-old SNR-derived organoids. GO terms were separately obtained for genes that show reduced (down) and increased (up) expression levels along the trajectories.

File Name: Supplementary Data 4
Description: The lists of human fetal brain region-specific and organoid cluster-specific genes used for the Jaccard similarity analysis presented in Fig. 4a.

File Name: Supplementary Data 5
Description: List of all cluster-specific genes detected in different clusters in 5-month-old SNR-derived organoids. Abbreviations: avg_logFC - Log fold-change of the average gene expression; pct.1 - the percentage of cells with detected gene expression in the target cluster; pct.2 - the percentage of cells with detected gene expression in the background cluster; p_val – unadjusted p-value; p_val_adj - p-value adjusted using the Bonferroni multiple comparison test.

File Name: Supplementary Data 6
Description: Electrophysiological properties of neurons recorded in 5-month-old SNR-derived organoid slices. Sheet1 - cells with APs for clustering: electrophysiological characteristics of individual neurons that were used for clustering in Fig. 9d. Detailed descriptions of how individual characteristics were obtained or calculated are provided in the materials and methods section.
Sheet 2 – summary of intrinsic properties of control and SHANK3-deficient neurons presented in Fig. 10 and Supplementary Fig. 20. Sheet 3 - summary of synaptic properties of neurons measured in slices of 5-month-old control SNR-derived organoids (Fig. 9). The exact p values reported in the table were determined using an unpaired two-sided t-test.
Abbreviations: Vrest – resting membrane potential; f_i_curve_slope – the slope of the frequency- current curve; ap_threshold – the threshold of 1st action potential; ap_width – the width of 1st action potential measured at the half-height from the threshold to the peak; ap_peak_to_threshold – amplitude of 1st action potential, from the threshold to the peak; ap_trough_5w_to_threshold - spike through voltage measured at 5 times spike width after the spike threshold; avg_rheobase_latency – average latency at the rheobase; and adapt_avg – average adaptation index.

File Name: Supplementary Data 7

Description: Lists of all differentially expressed genes in control and SHANK3-deficient organoids presented in Fig. 10j-k. Sheet 1 – SHANK3+/- (EYQ line, 3 organoids) vs. iCtrl (H9 line, 3 organoids); Sheet 2 – SHANK3+/- (engineered line [3 organoids] and patient line [3 organoids]) vs. control (H9 line [3 organoids] and iPSC control line [3 organoids]). Differentially expressed genes were identified using a 5% false discovery rate with DESeq2 version 1.30.1 (Love et al., Gen Biol 2014). DESeq2 uses a Wald test for significance testing. The exact Wald test p values and adjusted for multiple comparisons p values using Benjamini and Hochberg procedure (Benjamini and Hochberg, J R Stat Soc Ser B Methodol. 1995) are reported in the table.
