# Supplementary material for: Modeling human telencephalic development and autism-associated SHANK3 deficiency using organoids generated from single neural rosettes
Source: Nat Commun. 2022 Oct 6;13:5688. doi: 10.1038/s41467-022-33364-z (PMC9537523; doi:10.1038/s41467-022-33364-z)
Supplement: Supplementary file 11 — Reporting Summary [file 41467_2022_33364_MOESM11_ESM.pdf]

nature research

Corresponding author(s): Alex Shcheglovitov, PhD

Last updated by author(s): Aug 31, 2022

Reporting Summary

Nature Research wishes to improve the reproducibility of the work that we publish. This form provides structure for consistency and transparency in reporting. For further information on Nature Research policies, see our Editorial Policies and the Editorial Policy Checklist.

Statistics

For all statistical analyses, confirm that the following items are present in the figure legend, table legend, main text, or Methods section.

n/a	Confirmed	
☐	☒	The exact sample size (*n*) for each experimental group/condition, given as a discrete number and unit of measurement
☐	☒	A statement on whether measurements were taken from distinct samples or whether the same sample was measured repeatedly
☐	☒	The statistical test(s) used AND whether they are one- or two-sided<br>*Only common tests should be described solely by name; describe more complex techniques in the Methods section.*
☒	☐	A description of all covariates tested
☐	☒	A description of any assumptions or corrections, such as tests of normality and adjustment for multiple comparisons
☐	☒	A full description of the statistical parameters including central tendency (e.g. means) or other basic estimates (e.g. regression coefficient) AND variation (e.g. standard deviation) or associated estimates of uncertainty (e.g. confidence intervals)
☐	☒	For null hypothesis testing, the test statistic (e.g. *F*, *t*, *r*) with confidence intervals, effect sizes, degrees of freedom and *P* value noted<br>*Give P values as exact values whenever suitable.*
☒	☐	For Bayesian analysis, information on the choice of priors and Markov chain Monte Carlo settings
☒	☐	For hierarchical and complex designs, identification of the appropriate level for tests and full reporting of outcomes
☐	☒	Estimates of effect sizes (e.g. Cohen's *d*, Pearson's *r*), indicating how they were calculated

Our web collection on statistics for biologists contains articles on many of the points above.

Software and code

Policy information about availability of computer code

Data collection

pClamp 10 (Molecular Devices)
ZEN 2.3 SP1FP3 (black) (Zeiss)
NIS-Elements (Nikon)
Image Lab (Bio-Rad, 1708265)
Intan RHX Data Acquisition Software

Data analysis

Single-cell RNAseq analysis:
10X Genomics Cell Ranger 2.0
scater R package
Slingshot (v0.1.3) R package
DoubletFinder (v2.0) R package
Seurat (v2.3.4 and v3.1.0) R package
scran (v1.5) R package
DoubletDetection (v2.4) Python package
umap-learn (v0.3.0) Python package
Velocyto (v0.17.8) Python package
ToppGene Suite
Shiny single cell browser R package (https://github.com/yueqiw/shiny_cell_browser)

Bulk RNAseq analysis:
STAR v2.7.6a
clumpify v38.34
featureCounts v1.6.3
DESeq2 version 1.30.1

PANTHER 15.0

Electrophysiology analysis:
DataJoint (v0.10.0) Python package
stfio (v0.14) Python package
Allen SDK, 2015
Clampfit 10 software
Ephys Analysis scripts (https://github.com/yueqiw/ephys_analysis)

Imaging analysis:
Fiji (ImageJ2 version: 2.3.0/1.53f)
Volocity 5.1
CellProfiler 3.0

Quantification and statistics:
Microsoft Excel v16.52
GraphPad Prism 9
Matlab R2022a

For manuscripts utilizing custom algorithms or software that are central to the research but not yet described in published literature, software must be made available to editors and reviewers. We strongly encourage code deposition in a community repository (e.g. GitHub). See the Nature Research guidelines for submitting code & software for further information.

Data

Policy information about availability of data

All manuscripts must include a data availability statement. This statement should provide the following information, where applicable:
- Accession codes, unique identifiers, or web links for publicly available datasets
- A list of figures that have associated raw data
- A description of any restrictions on data availability

The data generated and analyzed in this study are included in this published article (and its supplementary information files) or available from the corresponding author upon reasonable request. Bulk and single-cell RNA sequencing data sets generated in this study were deposited in the Gene Expression Omnibus (GEO) of the National Center for Biotechnology Information (NCBI), under the following accession numbers: GSE210960 (https://www.ncbi.nlm.nih.gov/geo/query/acc.cgi?acc=GSE210960). Interactive visualization of our transcriptomic and electrophysiological data is provided in our online browser (UBrain Browser: http://organoid.chpc.utah.edu). Source data are provided as a Source Data file.

Field-specific reporting

Please select the one below that is the best fit for your research. If you are not sure, read the appropriate sections before making your selection.

☒ Life sciences ☐ Behavioural & social sciences ☐ Ecological, evolutionary & environmental sciences

For a reference copy of the document with all sections, see nature.com/documents/nr-reporting-summary-flat.pdf

Life sciences study design

All studies must disclose on these points even when the disclosure is negative.

Sample size	Sample sizes are reported in each figure legend. They were chosen based on the results of our previous studies with iPSC-derived neurons and pilot experiments with organoids. We also considered the sample sizes used in the recent studies on iPSC-derived organoids (Lancaster et al. Nature 2013; Pasca et al., Nat Methods 2015; Qian et al., Cell 2016; Quadrato Nature 2017; Velasco Nature 2019).
Data exclusions	Organoids were excluded from the experiments and analysis based on the following pre-established criteria:<br>1. The organoids failed to grow during the two weeks expansion phase in EGF/FGF<br>2. The organoids showed multiple clearly visible lumens at the time of embedding in Matrigel<br>3. Less than 50% recovered cells (trypan blue negative cells) after dissociation for single cell RNA sequencing experiments.<br>4. The single cell RNA sequencing sample contained less than 500 recovered cells after sequencing.<br>Cells were excluded from single cell RNA sequencing analysis based on the following criteria:<br>1. Cells with abnormally low or high amount of unique molecular identifier (UMI) counts, determined by the data distribution.<br>2. Cells with more than 6~10% of UMIs assigned to mitochondrial genes, as judged by the data distribution.<br>3. Putative multiplets as determined by DoubletFinder or DoubletDetection software package.<br>Cells were excluded from the analysis in electrophysiology experiments if they appeared as clear outliers and were identified as outliers by the ROUT method with Q=2%
Replication	Organoids were produced from 6 pluripotent stem cell lines through multiple round of differentiations. The reproducibility was assessed using single cell RNA sequencing on cells obtained from 6 different organoids produced from 4 stem cell lines in 3 different rounds of differentiations. Mutual index and z-score statistics were used to quantify the organoid to organoid reproducibility, as it was previously described by Velasco and colleagues (Velasco et al., Nature 2019).<br><br>All results were replicated on multiple organoids produced in several rounds of differentiations. The specific number of replicates used in different experiments is provided in each figure legends. All replication attempts were successful.

Randomization	No randomization was performed for stem cell lines to be used in different experiments. The names of the lines used in different experiments are described in the figure legends. Organoids/sections/ROIs/cells were randomly selected from wells/slides/sections/slices for data collection.
Blinding	The investigators were blinded for the genotype when the properties of control and SHANK3-deficient organoids were investigated and compared. The experiments were performed without blinding when no comparisons were performed. The analysis of mRNA sequencing results was performed on unblinded samples to identify the differentially expressed genes.

Reporting for specific materials, systems and methods

We require information from authors about some types of materials, experimental systems and methods used in many studies. Here, indicate whether each material, system or method listed is relevant to your study. If you are not sure if a list item applies to your research, read the appropriate section before selecting a response.

Materials & experimental systems

n/a	Involved in the study	
☐	☒	Antibodies
☐	☒	Eukaryotic cell lines
☒	☐	Palaeontology and archaeology
☒	☐	Animals and other organisms
☐	☒	Human research participants
☒	☐	Clinical data
☒	☐	Dual use research of concern

Methods

n/a	Involved in the study	
☒	☐	ChIP-seq
☒	☐	Flow cytometry
☒	☐	MRI-based neuroimaging

Antibodies

Antibodies used

Sox2 Abcam ab97959 Rb 1:500
Pax6 BioLegend/Covance 901301 (previously PRB-278P) Rb 1:250
Gsx2 Thermo Fisher PA5-35887 Rb 1:250
Nkx2.1 Millipore MAB5460 (clone 8G7-G3-1) Ms 1:200
N-Cadherin Abcam ab98952 Ms 1:500
Ki67 BD Biosciences 550609 Ms 1:100
Tuj1 Covance MMS-435P Ms 1:1000
Map2 Synaptic Systems 188 004 Gp 1:1000
Tbr2 EMD Millipore AB2283 Rb 1:300
Reelin MBL International D223-3 Ms 1:500
GABA Sigma A2052 Rb 1:500
GAD67 EMD Millipore MAB5406 Ms 1:500
Somatostatin Chemicon AB5494 Rt 1:500
Parvalbumin Swant PV 235 Ms 1:500
VIP Immunostar 20077 Rb 1:500
Calretinin Swant 7697 Rb 1:500
Calbindin (d28k clone) Swant 300 Ms 1:500
Darpp32 (h-3) Santa Cruz sc-271111 1:500
S100b Agilent Technologies Z031129-2 Rb 1:1000
GFAP Abcam ab4674 Ch 1:300
MBP Chemicon MAB386 Rt 1:500
O4 R&D Systems MAB1326-SP Ms 1:500
Tbr1 Abcam ab31940 Rb 1:500
Ctip2 (25B6 clone) Abcam ab18465 Rt 1:500
Satb2 (SATBA4B10 clone) Abcam ab51502 Ms 1:500
Cux1 Santa Cruz Biotechnology sc-13024 Rb 1:500
GFP Abcam ab13970 Ch 1:1000
Caspase-3 BD Pharmingen 559565 Rb 1:500
PH3 EMD Millipore 06-570 Rb 1:500
Foxp2 Abcam ab16046 Rb 1:500
Bassoon Enzo Life Sciences ADI-VAM-PS003-D Ms 1:500
Homer1 Synaptic Systems 160 004 Gp 1:500
Gephyrin Synaptic Systems 147 003 Rb 1:50
Vglut1 EMD Millipore AB5905 Gp 1:1000
Synapsin1 Synaptic Systems 106 001 Rb 1:500
PSD-95 (6G6-1C9 clone) Abcam ab2723 Ms 1:100
Shank1 Novus Biologicals NB300-167 Rb 1:100
Shank2 Synaptic Systems 162 202 Rb 1:200
Shank3 Synaptic Systems 162 304 Gp 1:200
Shank3 Synaptic Systems 162 302 Rb 1:100
PDGFR-β Santa Cruz Biotechnology sc-374573 Ms 1:100
FoxG1 (Bf1) Takara Bio M227 Ms 1:250
Fam107A Proteintech 12176-1-AP Rb 1:100

αSMA Thermo Fisher Scientific 710487 Rb 1:100

Alexa Fluor 405 goat anti-mouse IgG (H+L), Thermo Fisher Scientific, A31553
Alexa Fluor 488 goat anti-mouse IgG (H+L), Thermo Fisher Scientific, A11029
Alexa Fluor 488 goat anti-mouse IgM (μ chain), Thermo Fisher Scientific, A21042
Alexa Fluor 488 goat anti-rabbit IgG (H+L), Thermo Fisher Scientific, A11034
Alexa Fluor 488 goat anti-rat IgM (μ chain), Thermo Fisher Scientific, A21212
Alexa Fluor 488 Donkey anti Sheep IgG (H+L), Thermo Fisher Scientific, A11015
Alexa Fluor 568 goat anti-Rabbit IgG (H+L), ThermoFisher Scientific, A11036
Alexa Fluor 594 goat anti-guinea pig IgG (H+L), Thermo Fisher Scientific, A11076
Alexa Fluor 594 goat anti-mouse IgG (H+L), Thermo Fisher Scientific, A11005
Alexa Fluor 594 goat anti-rat IgM (μ chain), Thermo Fisher Scientific, A21213
Alexa Fluor 647 goat anti-mouse IgG (H+L), Thermo Fisher Scientific, A21235
Alexa Fluor 647 goat anti-guinea pig IgG (H+L), Thermo Fisher Scientific, A21450
Alexa Fluor 647 goat anti-Rabbit IgG (H+L), Thermo Fisher Scientific, A21245
Alexa Fluor 647 goat anti-Rat IgG (H+L), Thermo Fisher Scientific, A21247
Streptavidin, Alexa Fluor 647 conjugate, Thermo Fisher Scientific, S21374

Validation

The primary antibodies used in this study have been validated and used in many previous studies on stem cell-derived brain organoids and fetal human brain tissue (Lancaster et al. Nature 2013; Pasca et al., Nat Methods 2015; Qian et al., Cell 2016; Quadrato Nature 2017; Velasco Nature 2019).
The following antibodies were validated in this study using embryonic mouse brain tissue:
Tbr2 EMD Millipore AB2283 Rb 1:300
Nkx2.1 (clone 8G7-G3-1) Millipore MAB5460 Ms 1:200

For Nkx2.1 validation, we performed immunostaining on coronal brain sections obtained from a conditional Rosa_tdTomato/Nkx2.1_Cre mouse at E14.5. Nkx2.1 expression overlapped with tdTomato expression in the MGE (Figure S4).

For Tbr2 validation, we performed immunostaining on coronal brain sections obtained from an embryonic mouse brain at E14.5. Tbr2-expressing cells demonstrated predicted distribution in the pallial subventricular zone (Figure S7).

Eukaryotic cell lines

Policy information about cell lines

Cell line source(s)

H9 [WiCell],
2242-5 [Dolmetsch lab, Stanford University]
GM07492 [Ernst lab, McGill University (Bell et al., 2018)]
EYQ2-20 [generated in the Shcheglovitov lab, (Chiola at al., 2021)]
EP2-15 [generated in the Shcheglovitov lab]
7349.3 [Dolmetsch lab, Stanford University]

Authentication

iPSC lines were tested for the expression of pluripotency markers using qRT-PCR and immunostainings. Conventional PCR and gel electrophoresis with the set of primers described in the materials and methods and in Chiola et al., 2012 were used to confirm the genotypes of engineered lines.

Mycoplasma contamination

All stem cell lines were periodically tested for mycoplasma contamination using PCR Mycoplasma test kit (PromoCell, PK-CA91-1048). We mycoplasma free cultures were used in the study.

Commonly misidentified lines
(See ICLAC register)

No such lines were used in this study

Human research participants

Policy information about studies involving human research participants

Population characteristics

N/A

Recruitment

Participants were recruited at Stanford University, de-identified iPSC lines were transfered to the University of Utah under an MTA agreement

Ethics oversight

Postmortem human brain specimens were obtained from University of Cambridge, UK upon pregnancy terminations. All procedures were approved by the research ethical committees and research services division of the University of Cambridge and Addenbrooke's Hospital in Cambridge (protocol 96/85, approved by Health Research Authority, Committee East of England—Cambridge Central in 1996 and with subsequent amendments, with the latest approved November 2017). Tissue was handled in accordance with ethical guidelines and regulations for the research use of human brain tissue set forth by the National Institute of Health (NIH) (http://bioethics.od.nih.gov/humantissue.html) and the World Medical Association Declaration of Helsinki (http://www.wma.net/en/30publications/10policies/b3/index.html).

Note that full information on the approval of the study protocol must also be provided in the manuscript.
